# Repeatable Genomic Outcomes Along the Speciation Continuum: Insights From Pine Hybrid Zones (Genus *Pinus*)

**DOI:** 10.1111/mec.70137

**Published:** 2025-10-13

**Authors:** Bartosz Łabiszak, Sebastian Szczepański, Witold Wachowiak

**Affiliations:** ^1^ Department of Plant Ecology and Environmental Protection, Institute of Environmental Biology Adam Mickiewicz University in Poznań Poznań Poland; ^2^ Department of Genetics and Environmental Interactions Institute of Dendrology, Polish Academy of Sciences Kórnik Poland

**Keywords:** contact zones, hybridization, interspecific gene flow, introgression, SNP genotyping, speciation

## Abstract

Hybridization is a widespread evolutionary process and a key source of evolutionary novelty. Despite intensive study, the extent to which hybridization is deterministic and repeatable, particularly in recurrent contact events involving the same species under varying ecological conditions, remains unclear. Here, we investigated three replicated contact zones between Scots pine ( *Pinus sylvestris* ) and dwarf mountain pine ( *Pinus mugo* ) in Central Europe: two occurring in peatland habitats and one in a contrasting sandstone outcrop. Using genome‐wide SNP genotyping of over 1300 individuals, we analysed genomic structure, diversity, and ancestry patterns across these zones. All sites revealed pervasive hybridization, dominated by later‐generation hybrids and a notable scarcity of pure *P. mugo* . Across environments, hybrid populations exhibited strikingly consistent genomic compositions, with asymmetric introgression strongly biased toward *P. mugo* ancestry, suggesting that hybrid genome structure may follow predictable patterns under similar ecological conditions and could be shaped by cytonuclear incompatibilities. Nonetheless, we also detected site‐specific differences in hybrid diversity and phenotype, highlighting the influence of local environmental selection on shared hybrid genomic backgrounds. We provide genomic evidence that *Pinus uliginosa*, a morphologically distinct peat bog pine traditionally regarded as a relict and endangered species is instead a partially stabilised hybrid lineage. Its genome reflects incomplete hybridization and ecological filtering, yet it lacks sufficient genetic divergence to be recognised as a distinct species. Together, these results provide evidence for the repeatability of hybridization processes, which result in the formation of phenotypes reflecting a species continuum subjected to strong environmental pressures. The findings support the simplification of taxonomic nomenclature within the *Pinus mugo* complex, informing adaptive conservation strategies and the genetic management of hybrid lineages.

## 1 Introduction

Speciation is a fundamental evolutionary process underlying the remarkable diversity of life observed on Earth. The fossil record provides extensive evidence of the gradual and cumulative nature of this process (Benton and Pearson 2001), typically characterised by increasing genetic divergence and reproductive isolation arising through prolonged geographic or ecological separation (Coyne et al. 2004; Sobel et al. 2010; Dieckmann and Doebeli 1999; Mallet, et al. 2009). Nevertheless, clear boundaries demarcating stages of speciation rarely exist, and the process often remains incomplete for extended periods, allowing ongoing gene flow between partially diverged lineages. Hybridization, in such contexts, encompasses a broad spectrum of evolutionary outcomes. These may include negative effects such as hybrid breakdown, reduced fitness, or limited backcrossing success (Coyne et al. 2004). Alternatively, they may include processes that promote diversification through introgression, the formation of hybrid swarms, and hybrid speciation. These phenomena are now collectively interpreted within the broader framework of the speciation continuum (Shaw and Mullen 2014).

Pines (*Pinus* spp.) are particularly useful models for investigating the processes of divergence and hybridization along this continuum. Within gymnosperms, pines constitute a diverse group comprising approximately 120 species naturally distributed throughout the Northern Hemisphere, inhabiting environments ranging from Siberian boreal forests to tropical islands in the Caribbean and Southeast Asia (Farjon 2010, 2018a). Due to overlapping geographical ranges and generally weak reproductive isolation, pine species frequently hybridise, producing viable offspring (Białobok et al. 1993; Critchfield et al. 1975; Mirov 1967). Such hybridization is well‐documented among conifers, often resulting in species complexes with indistinct boundaries, including rare cases of ancient homoploid hybrid speciation. Ecological perturbations, including those induced by climate change or human activities, can increase the likelihood of hybridization by facilitating secondary contacts, eliminating ecological barriers, or altering niche availability. They may also reduce hybridization by disrupting reproductive synchrony or reinforcing prezygotic isolation through changes in phenology (Grabenstein and Taylor 2018; Vonlanthen et al. 2012; Harrison and Larson 2014). Depending on the context, novel ecological niches arising from environmental disturbances can either promote the establishment and persistence of hybrid populations or act as filters that limit hybrid success, potentially influencing whether hybrid lineages persist and diversify (Schumer et al. 2014; Moran et al. 2021; Rieseberg et al. 2003). Understanding these evolutionary dynamics is increasingly crucial given that hybridization events are expected to rise due to climate‐driven range shifts and anthropogenic introductions (Grabenstein and Taylor 2018; Chunco 2014). Furthermore, environmental changes often weaken prezygotic barriers, fostering hybridization and creating new habitats suitable for hybrid establishment (Grabenstein and Taylor 2018; Taylor and Larson 2019). However, for many hybridising species, critical questions remain unanswered: will hybrids with consistent genomic compositions repeatedly emerge from contact zones involving identical parental species under varying ecological conditions? Moreover, how do environmental differences influence hybrid genetic diversity and patterns of introgression?

Here, we address these questions by investigating closely related European hard pines, Scots pine ( *Pinus sylvestris* L.) and members of the dwarf mountain pine ( *Pinus mugo* Turra) species complex. Scots pine is a widespread foundation species, naturally distributed across diverse ecosystems in Europe and Asia, including boreal peatlands, dry Caucasian regions, and harsh climates in eastern Siberia (Farjon 2018a; Białobok et al. 1993; Carlisle and Brown 1968). Its distribution is mostly allopatric relative to taxa within the *P. mugo* complex, which primarily inhabit alpine and subalpine environments, including peat bogs (Hamernik and Musil 2007; Christensen 2008; Boratyńska et al. 2015). Taxonomic delineation within the *P. mugo* complex remains challenging due to ambiguous species boundaries and ongoing debate over genetic differentiation, particularly among taxa such as *P. mugo* Turra, *P. uncinata* Ramond, and *P. uliginosa* Neumann (Christensen 2008; Businský and Kirschner 2010; Neumann 1837). Several sympatric populations involving *P. sylvestris* and members of the *P. mugo* complex have been documented in post‐glacial peat bogs harbouring relict populations of *P. mugo* , notably in the Sudetes, Orawsko‐Nowotarska Valley, the Alps, and elsewhere (Klobucnik et al. 2022; Heuertz et al. 2010; Boratynska, et al. 2010).

In this study, we employed molecular methods to characterise genomic composition and hybridization dynamics within these pine contact zones, aiming to clarify the role of hybridization and introgression in species divergence. Specifically, we analysed thousands of nuclear single nucleotide polymorphisms (SNPs), predominantly from coding genomic regions, in a large dataset comprising over 1300 individuals sampled from allopatric stands of *P. sylvestris* , *P. mugo* , and their hybrid populations. Additionally, we included a reference stand of peat‐bog pine ( *P. uliginosa* ) from its *locus classicus*, as individuals of such phenotype have been observed in some of the investigated contact zones. This approach allowed us to resolve recent findings indicating extensive reticulation and ongoing gene flow between *P. uliginosa* and related taxa (Łabiszak and Wachowiak 2021; Sikora and Celinski 2024). By examining populations varying in phenotypic composition and geographic context, we assessed how hybridization influences genetic divergence and introgression patterns, and explored whether hybrid populations represent partially stabilised hybrid lineages with distinct ecological characteristics. We also clarified the taxonomic and evolutionary status of *P. uliginosa* , currently recognised as a relict and endangered taxon, providing valuable insights toward adaptive conservation and effective management of genetic resources.

## 2 Material and Methods

### 2.1 The Study Area and Sampling

**FIGURE 1:**
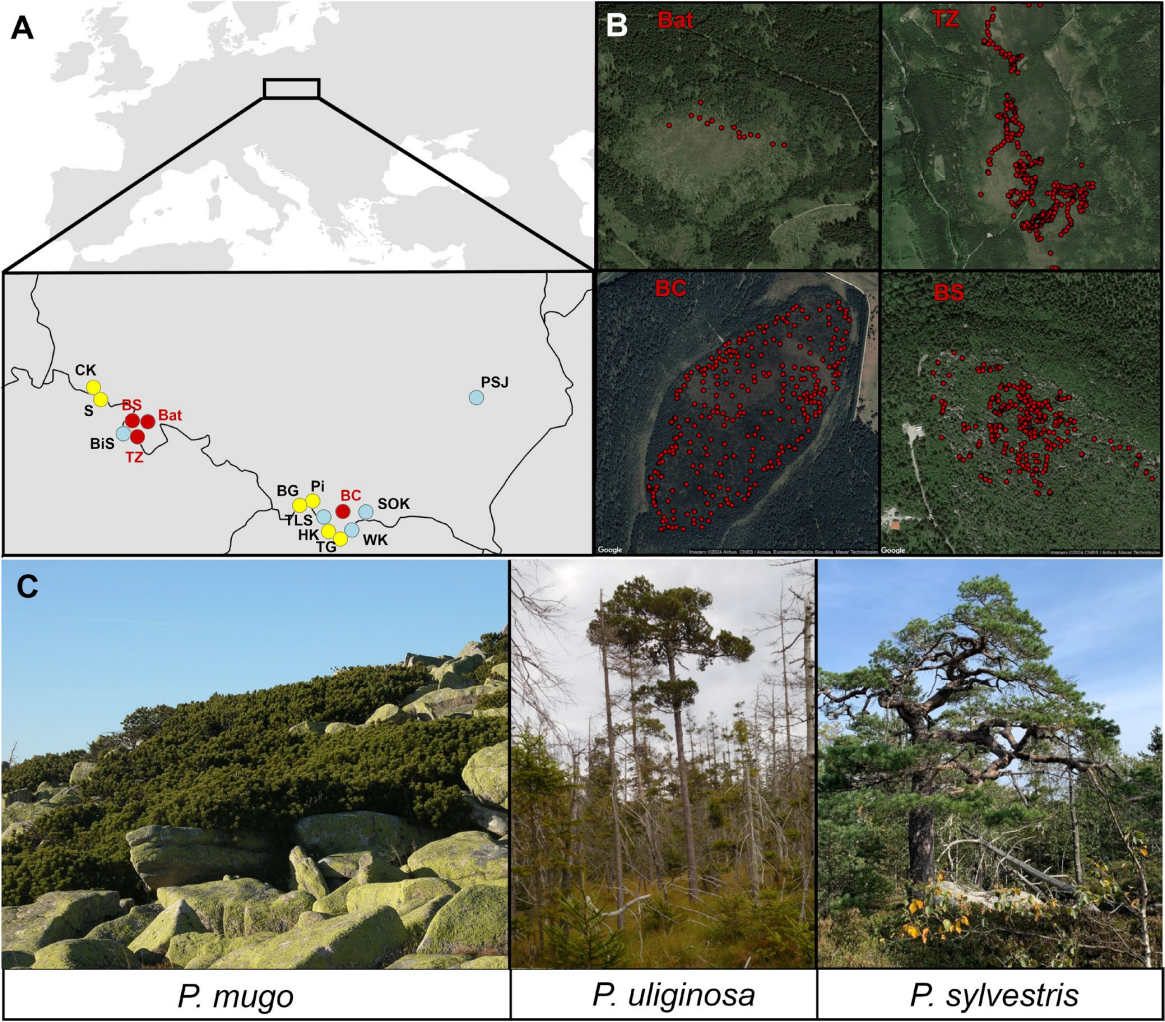
Geographic location and morphological overview of studied pines population. (A) Map indicating locations of studied populations in Central Europe, with populations colour‐coded by species. Allopatric populations of *P. mugo* and *P. sylvestris* are yellow and blue, respectively, *P. uliginosa* stand (Bat) and three contact zones of different pine composition (TZ, BC, BS) are coloured in red. (B) Map of spatial distribution patterns within three studied contact zones and Bat stand. (C) Representative individuals displaying characteristic morphological features of three studied pines: *Pinus mugo* , *Pinus uliginosa* and *Pinus sylvestris* .

This study focused on three distinct hybrid zones of pine species located in southern Poland, characterised by various sizes and species compositions. Additionally, 11 allopatric reference populations of parental species were analysed, including five populations of Scots pine ( *Pinus sylvestris* L.) and six of dwarf mountain pine ( *P. mugo* T.) (Figure 1A, Table S1). At the studied area, the current ranges of *P. sylvestris* and *P. mugo* are largely non‐overlapping due to altitudinal isolation (Figure S1). Scots pine typically grows at elevations from sea level up to 1000 m above sea level (asl), while dwarf mountain pine is found at elevations between 1100 and 2200 m asl. Furthermore, in mountainous regions, Scots pine has been largely displaced by Norway spruce ( *Picea abies* ) and other conifers, with only a few natural relict populations remaining in the Stołowe Mountains and the Tatra Mountains (i.e., Białe Skały, Wielkie Koryciska, Łysa Skałka) included in this study (Boratyński 1993; Zwijacz‐Kozica 1998). Furthermore, we included the population of peat‐bog pine (*Pinus uliginosa* N.) from Wielkie Torfowisko Batorowskie (Bat), where the species was first described in the nineteenth century (Neumann 1837; Wimmer 1837). This population is located in the Stołowe Mountains on a 35 ha mountainous peat bog that formed around 10,000 years ago in a shallow sedimentary basin. The remnants of *P. uliginosa* (currently no more than 100 individuals) grow in sphagnum spruce forests with birch, but no other pines (Woronko 2002; Danielewicz and Zieliński 2000; Marek 1988).

The analysed contact zones include Błędne Skały (BS) in the Stołowe Mountains, Torfowisko pod Zieleńcem (TZ) in the Bystrzyckie Mountains, and Bór na Czerwonem Reserve (BC) in the Nowotarska Valley (Figure 1C). The first one, Błędne Skały (BS), features pines growing atop sandstone maze‐like rock formations in hard‐to‐access locations with a thin layer of soil. This mixed population is considered a relic from the Boreal period of the Holocene (9000–8000 years BP) (Sobierajska et al. 2020; Boratyński 1978). It consists of scattered pine individuals exhibiting *P. sylvestris* and *P. uliginosa* phenotypes, a few *P. mugo* ‐like individuals, and numerous intermediates considered as putative hybrids. This population is surrounded by extensive Norway spruce ( *Picea abies* ) forests, isolating it from direct contact with other Scots pine populations. The nearest natural population of *P. mugo* is in the Karkonosze Mountains, approximately 70 km away (Figure 1A, Figure S1).

The second contact zone, Torfowisko pod Zieleńcem (TZ), is a peat bog, dating from the Boreal period, that is one of the largest (~200 ha) raised bogs in the Sudety Mountains region (Madeyska 2005). The area is divided into two major sections, each with distinct plant communities: the northern part is a typical open raised bog, while the southern part is a transitional bog interspersed with fragments of spruce forest. This bog supports many relict species from the last glaciation, such as dwarf birch ( *Betula nana* ), bog rosemary ( *Andromeda polifolia* ), and bog cranberry ( *Vaccinium oxycoccos* ) (Madeyska 2005). The peat bog harbours a complex pine population, with a few individuals of *P. sylvestris*, dense stands of *P. mugo‐like* shrubs, and trees with phenotypes of *P. uliginosa* . In addition, there are individuals that show a range of growth forms, from polycormic structures to more tree‐like forms, and exhibit highly variable appearances.

The third contact zone, Bór na Czerwonem Reserve (BC), is located approximately 25 km north of the Tatra Mountains. This population grows on a peat bog formed around 10,000 years BP and is part of a larger complex of peat bogs in the Orawa–Nowy Targ Basin, which includes 33 individual sites of varying sizes, covering nearly 200 km^2^ (Łajczak 2009). In this region, pines are represented by *P. sylvestris* , *P. mugo* , and their putative hybrids. Notably, in contrast to BS and TZ contact zones, which are not directly influenced by nearby pure parental species stands, the BC population is surrounded by managed Scots pine forest. However, morphological forms resembling *P. uliginosa* have not been reported there (Bobowicz and Danielewicz 2014; Bączkiewicz 1995).

The samples were collected between 2021 and 2023, totaling 1345 individuals. This included 1020 individuals from contact zones (300 from BS, 420 from TZ, and 300 from BC) and 325 individuals from reference populations, with an average of 28 individuals per population. The sampling was carried out under permits from the Polish Ministry of Climate and Environment (DOP‐WPN.61.116.2021.MGr; DOP‐WOPPN.61.35.2022.WH) and the Polish State Forests (ZG.7021.2.2021). Individuals from contact zones were classified based on their phenotype and morphological traits (Boratyńska et al. 2021; Boratyńska and Boratyński 2007) as one of the pine species or marked as hybrids (Supporting Information S1). Total genomic DNA was extracted from fresh needle tissue using the Genomic Mini AX Plant extraction kit (A&A Biotechnology, Poland). DNA concentration was assessed with a Qubit 4 fluorometer using the Broad Range (BR) Assay Kit, and samples were diluted to a working concentration of 40 ng/μL.

### 2.2 SNP Genotyping and Data Processing

Single nucleotide polymorphism (SNP) data were generated using a custom‐made Axiom PineGAP SNP array (Affymetrix, Thermo Fisher Scientific, Santa Clara, CA, USA) by the Bristol Genomics Facility (Bristol, UK). This array was designed specifically to target polymorphism in the analysed pine species and includes 49,829 SNP markers, mainly from functional regions of transcriptomes (Wachowiak et al. 2015) and candidate genes from pine resequencing studies (Perry et al. 2020). Genotypes were called using the Axiom Analysis Suite software (Applied Biosystems, Waltham, MA, USA), following the manufacturer's protocol with adjusted quality thresholds to increase stringency: QC call_rate was set to 95%, average cut‐rate for passing samples to 95%, and Cr‐cutoff to 95%. Those parameters control genotyping quality and SNP filtering. QC call_rate sets the minimum per‐sample call rate, ensuring only high‐quality samples are retained. Average cut‐rate defines the acceptable mean call rate across probes to filter out low‐quality SNPs. Cr‐cutoff applies a SNP‐level threshold, removing variants with insufficient call rates. Genotypes classified as “Best and Recommended” were retained for downstream filtering. Subsequent quality control steps were performed in PLINK v1.07 and included: (i) removal of SNPs with BLAST matches to the *P. taeda* mitochondrial genome (NC_039746.1) or *P. sylvestris* chloroplast genome (NC_035069.1); (ii) exclusion of loci or individuals with > 5% missing data; and (iii) filtering of SNPs with minor allele frequency (MAF) < 0.05. We applied a conservative minor allele frequency threshold of 5% to minimise the influence of rare variants on population structure inference, particularly in model‐based approaches (Linck and Battey 2019). This filtering aimed to emphasise deeper genomic signals associated with hybridization and introgression between *P. sylvestris* and *P. mugo* . Consistent ancestry estimates across PCA, LEA, and hybrid index analyses (see results below) confirmed that our results were robust to the exclusion of low‐frequency variants. Furthermore, linkage disequilibrium pruning (*r* ^2^ > 0.7) was applied to reduce marker redundancy prior to structure and clustering analyses.

Additionally, we utilised information from the species diagnostic fragment of the *trn*L‐*trn*F intergenic region of chloroplast DNA, which distinguishes the paternally transmitted chloroplast genome of the species. There are two possible variants at this locus: variant *cp*A, which is characteristic of pines within the *Pinus mugo* complex (including *P. mugo* and *P. uliginosa* ), and variant *cp*C, which is observed in *Pinus sylvestris* (Wachowiak et al. 2014). The markers were generated following the protocol described in (Szczepański et al. 2023).

### 2.3 Genetic Structure

The structure of the population in hybrid zones was examined to understand the degree of admixture and genetic composition of the pines from hybrid zones. We used a robust method, capable of handling large genomic datasets, based on Latent Factor Models implemented in the *LEA* R package (Frichot and François 2015). We tested for ancestral clusters ranging from *K* = 1 to *K* = 10, using 10 replications for each K, to determine cross‐entropy (Frichot et al. 2014). The graphical illustration of individual ancestry coefficients was plotted using the POPHELPER Structure Web App v1.0.10 (Francis 2017). Based on the estimates of the ancestry coefficient expressed as Q‐scores, we classified our sampled trees into parental species ( *Pinus sylvestris* , *Pinus mugo* ) and hybrids (see *Results* section for explanation of the lack of *P. uliginosa* as parental species). The classification of hybrids was further refined into putative first‐generation hybrids (F1) and advanced backcross generations, such as H_PS (hybrids with a majority of *P. sylvestris* ancestry) and H_PM (hybrids with a majority of *P. mugo* ancestry) depending on their ancestry proportions (Supporting Information S1). We defined individuals as pure species if they exhibited genome‐wide admixture proportions of less than 3% admixture from the other species, early generation hybrids (putative F1) as individuals with approximately 50% ancestry from both clusters (45%–55%), and finally advanced backcross individuals with ancestry proportions showing significant admixture from one parent species (55%–97%). The proportion of hybrids was assessed separately within each hybrid zone to detect local variation in hybridization rates. To further investigate the extent and direction of hybridization across the populations, we contrasted ancestry coefficients with information from the diagnostic *cp*DNA marker (*trn*L‐*trn*F), distinguishing the paternally inherited chloroplast genome between species (Supporting Information S1). This combined approach allowed us to infer not only the degree of genetic mixing but also the direction of gene flow in the hybrid zones.

To assess the distribution of genetic variance and further corroborate the results of the LEA analysis, we performed principal component analysis (PCA). We ran sets of nested PCAs at the individual level using the *adegenet* and *ade4* R package (Jombart and Ahmed 2011; Jombart 2008), starting with the complete SNP dataset across all sampled populations to characterize the broad‐scale genetic structure of the pines. Missing genotype data were handled using mean imputation across individuals for each SNP. PCA was performed using the dudi.pca() function, with genotypes coded as allele counts (0, 1, 2), mean‐centered, and scaled to unit variance. Prior to the analysis, we removed loci with MAF < 5% to limit the disproportionate influence of rare variants (Patterson et al. 2006). Subsequently, we performed separate PCA analyses for each contact zone, using reference *P. mugo* and *P. sylvestris* to contrast the hybrid pines along the principal component axes. We further evaluated the discrimination power of this analysis by looking at the patterns found within the putative parental species alone.

### 2.4 Genetic Diversity Within Contact Zones

Based on the results of genetic structure analysis, we calculated observed (Ho) and expected heterozygosity (He), allele richness, and fixation index F_IS_ in each population and separately for each species group, including *Pinus sylvestris* , *P. mugo* , and their hybrids using the *hierfstat* package in R (Goudet 2005; R Core Team 2025). When considering the population‐level genetic diversity measures, in addition to allopatric populations of parental species, we included individuals genetically assigned as pure Scots pines within contact zones as separate regional populations (e.g., PS_TZ for pure *P. sylvestris* from the TZ contact zone). Hybrid individuals were grouped into populations based on their geographic origin and predominant ancestry: for instance, TZ_H_PS refers to hybrids from TZ with predominant *P. sylvestris* ancestry, while BS_H_PM indicates hybrids from BS with predominant *P. mugo* ancestry. We also ran the analysis including putative F1 hybrids within the contact zone as an additional population group. Based on our results, we decided not to assign the 30 individuals from TZ as pure *P. mugo* but to include them in the hybrid group when assessing the overall diversity (see *Results* and *Discussion* sections for additional information). To test for significant differences in genetic diversity between species groups, we performed an analysis of variance (ANOVA). Post hoc pairwise comparisons were performed using Tukey's honestly significant difference (HSD) test to determine which species groups differed significantly. Genetic differentiation among populations and overall between pure species and hybrids was determined using the pairwise F_ST_ calculated according to Weir and Cockerham (Weir and Cockerham 1984) in *adegenet* (Jombart and Ahmed 2011; Jombart 2008) and visualised with the *corrplot* package (Wei and Simko 2017). In addition, we conducted a hierarchical F‐statistics analysis using the *hierfstat* package in R, grouping individuals first by ancestry class (PS, PM, H_PS, H_PM) and then by population of origin.

### 2.5 Hybrid Index and Patterns of Introgression

As simulation studies have shown, STRUCTURE‐like analysis and PCA alone often fail to discriminate between two distinct demographic histories: admixture and isolation by distance (Wiens and Colella 2024a). To overcome this limitation, we employed two complementary methods. First, we calculated a hybrid index using the *gghybrid* package in R that employs a Bayesian Markov chain Monte Carlo (MCMC) approach for hybrid index estimation. We retained only ancestry‐informative markers (AIMs), those SNPs for which the smaller of the two parental minor allele frequencies is less than 10%. This allowed us to retain SNPs that are not fixed for alternate alleles in parental populations, providing for denser sampling of the entire genome in estimating the hybrid index. Furthermore, we evaluated the distribution of these AIMs in hybrids to determine whether AIMs tend to exhibit a bias toward one parent. We extended this analysis to different hybrid zones, providing insight into the dynamics of introgression in hybrid populations.

Next, we supplemented the hybrid index data using the *triangular* R package (Wiens and Colella 2024b) which integrates genotype data to calculate interclass heterozygosity. This metric helps identify hybrid classes and provides evidence of admixture through triangular plots. Individuals were classified into six genotypic categories according to Fitzpatrick (Fitzpatrick 2012). Additionally, we calculated the correlation between the hybrid index and ancestry coefficients to test whether inference based only on later could potentially introduce biased results.

To infer the optimal number of admixture events in our hybrid populations, we applied a composite likelihood approach implemented in TreeMix v1.13 (Pickrell and Pritchard 2012). First, we ran TreeMix with 500 bootstraps for each migration event (M) tested, in the range of 1–10. The optimal number of migration edges was determined using the *OptM* package in R (Fitak 2021). We evaluated TreeMix outputs from multiple runs, based on Evanno‐like statistics that calculate the second‐order rate of change in log‐likelihoods to select the most appropriate number of migration edges. A consensus maximum likelihood tree with the optimum number of migration events was then created. The residual covariance matrix was estimated to identify pairs of populations with significant deviations from the drift‐only model. The calculations and visualisations were done following the TreeMix pipeline script (Dahms 2021).

To verify the occurrence of admixture events and identify potential sources of introgression within the contact zones, we calculated the f3 statistic using the *AdmixTools2* R package (Maier and Patterson 2024). We applied the f3 test in the form of (X; A, B), where A and B represented the parental populations (pure *P. mugo* and *P. sylvestris* ), and X represented our hybrid populations (e.g., TZ). This approach allowed us to determine whether hybrid populations were the result of gene flow between the parental species or if there were contributions from other, unrecognised source populations. Finally, we looked at how genetic composition matched the phenotypes of hybrids across the contact zones.

## 3 Results

### 3.1 Genetic Composition of Pines From Contact Zones

Of the 49,829 SNPs on the PineGAP array, 15,627 were initially classified as “Best and Recommended” by the Axiom Analysis Suite. Following successive quality control steps including removal of organellar matches, low‐frequency alleles, and loci with excessive missing data, 8887 nuclear SNPs were retained. After LD pruning, a final panel of 7390 high‐confidence SNPs was used for downstream analyses. This corresponds to a conversion rate of approximately 31%, which is slightly lower than the 41.7% reported during array validation (Perry et al. 2020). However, such reductions are in line with those reported for other Axiom arrays developed for conifers, where 30%–40% retention is typical following similar filtering procedures (Jackson et al. 2022; Howe et al. 2020; Olsson et al. 2025). The retained SNPs were broadly distributed across the transcriptome, with most mapping to unique contigs (Figure S2), reflecting the original array design strategy of maximizing coverage of independent, coding regions. High‐quality genotype data were obtained for 1323 individuals from both contact zones and reference populations (Figure 1, Table S1), with a mean genotyping success rate of 98.3%.

**FIGURE 2:**
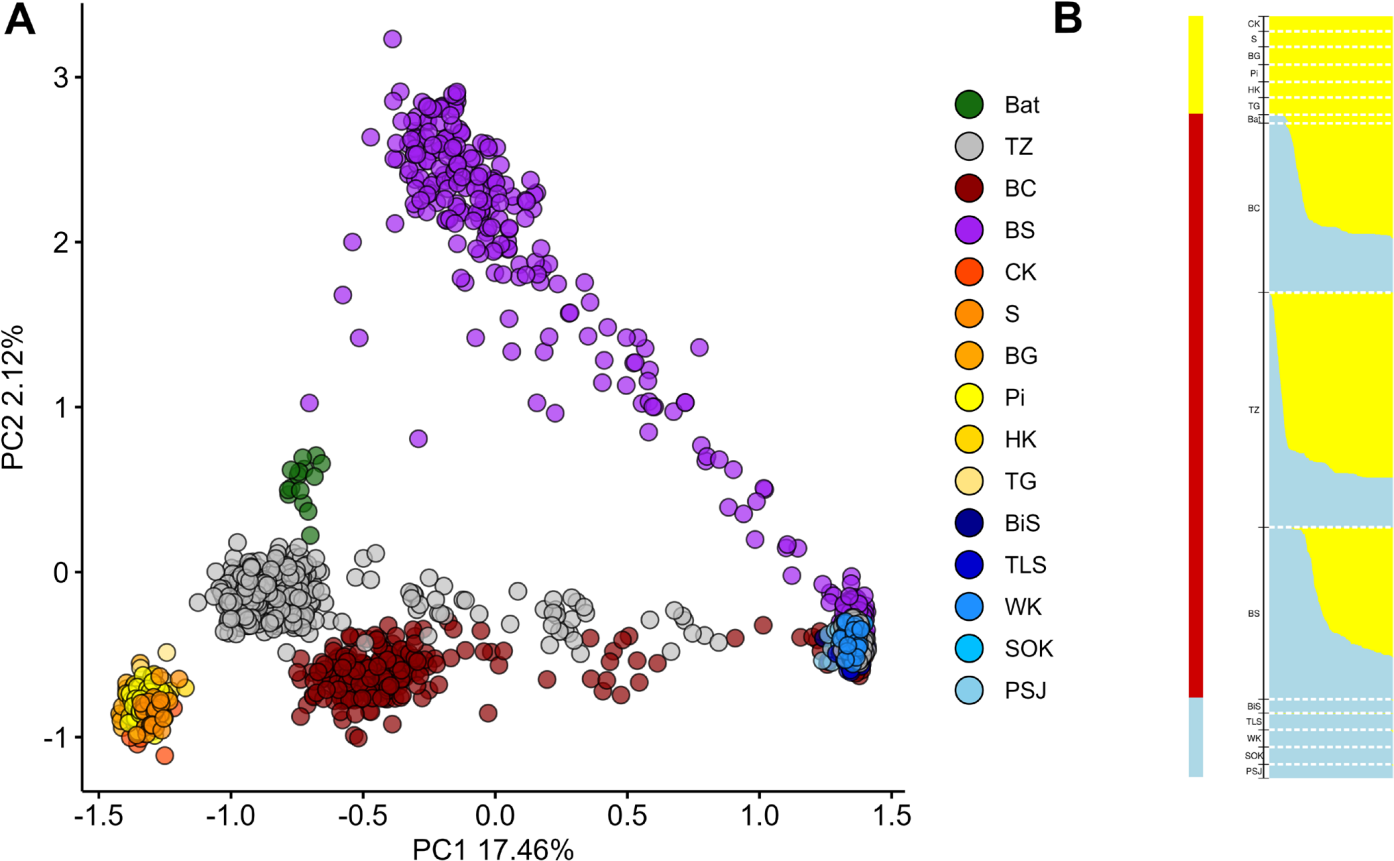
Genetic structure of pines across speciation continuum. (A) Principal component analysis (PCA) of all 1323 individuals projected along PC1 and PC2. Individual trees are consistently colour‐coded according to their population of origin. Allopatric populations of *P. mugo* and *P. sylvestris* are shown using gradients of their respective primary colours (as in Figure 1), while contact zone populations are represented by distinct colours. (B) Ancestry proportions of individual trees from allopatric and contact zone populations, inferred using LEA for *K* = 2 ‐ the most likely number of genetic clusters, as determined by the cross‐entropy criterion (see Figure S2). Bars represent individual trees, with ancestry components coloured according to *P. mugo* (yellow) and *P. sylvestris* (blue). Admixed populations are outlined with a red line.

An individual ancestry coefficient analysis performed in LEA identified two main genetic groups (*K* = 2, Figure 2, Figure S3) representing the ancestry of *P. sylvestris* and *P. mugo* . There was no indication of a separate genetic cluster for *P. uliginosa* from its *locus classicus* at Bat or in any other population in the contact zone. Instead, we observed mixed ancestry, as evidenced by Q‐scores ranging from 0.03 – 0.97 among individuals in the contact zones, while individuals from allopatric populations were classified as pure *P. mugo* or *P. sylvestris* . Interestingly, across all contact zones, some individuals were classified as pure *P. sylvestris* , while no individuals were categorized as pure *P. mugo* , except in the TZ population, where 30 individuals exhibited pure *P. mugo* ancestry (see Results Section 3.3).

This grouping was further supported by principal component analysis (PCA) revealing clear genetic structuring between *Pinus sylvestris* and *Pinus mugo* , with individuals from allopatric populations of each species clustering distinctly along the PC1 (Figure 2). The population structure within the allopatric populations of each species appears weak overall (Figure S4), although there are some signals of differentiation. In particular, in Scots pine ( *P. sylvestris* ), a slight divergence is observed in populations from the Tatra Mountains (WK, TLS), while in dwarf mountain pine ( *P. mugo* ), a distinct separation exists between populations from the Karkonosze Mountains (CK, S) and those from more eastern mountain ranges (populations BG, HK, Pi, and TG). Interestingly, individuals from contact zones do not form a discrete cluster of their own. Instead, they spread across the genetic space between the *P. sylvestris* and *P. mugo* clusters, highlighting a gradient of genetic admixture, although they are mostly skewed toward the *P. mugo* cluster (Figure 2, Figure S5). This first principal component, which explains 17.5% of genetic variation, captures the main axis of genetic variation, underscoring the divergence between the two species. Meanwhile, PC2, which explains only 2.1% of the variation, may correspond to subtler population‐level differences. Notably, the BS contact zone population displayed the most divergence compared to other sympatric populations.

### 3.2 Genetic Diversity

**FIGURE 3:**
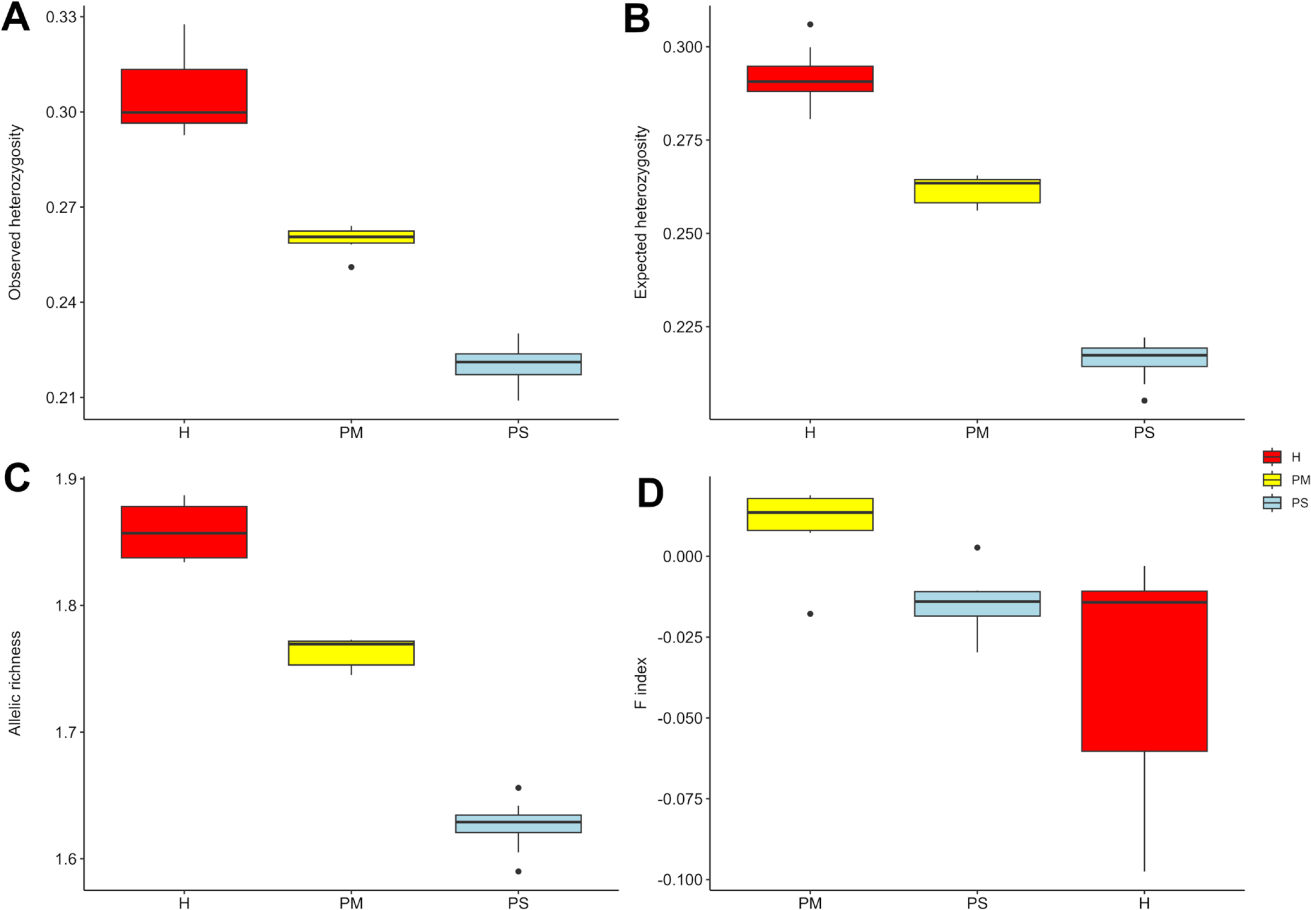
Genetic diversity levels in studied pines. Boxplots comparing mean values of: (A) observed heterozygosity, (B) expected heterozygosity, (C) allelic richness, and (D) fixation index (F_IS_) among individuals grouped by ancestry class: Hybrids (H), pure *Pinus mugo* (PM), and pure *Pinus sylvestris* (PS). Ancestry classes are colour‐coded as in Figure 2.

The analysis of genetic diversity patterns shows consistently that hybridization enhances genetic variation within hybrid zones. Genetic diversity expressed as heterozygosity (both observed and expected) and allelic richness is significantly higher in hybrids compared to both pure species (Figure 3, Table S2, Table S3). According to expectations, this effect was especially pronounced in putative F1 hybrids, as these individuals had the highest genetic diversity among all the studied pines (Figure S6, Figure S7). Hybridization had also an effect on inbreeding values, as indicated by the mean value of the fixation index F_IS,_ reduced close to zero in all hybrids (F_IS_ ~ −0.02), and having significantly lower values for putative F1 = −0.1. However, when comparing the inbreeding coefficient between hybrids and parental species, the only statistically significant difference was between hybrids and *P. mugo* , where a slightly positive inbreeding coefficient was reported. Interestingly, the distribution of the inbreeding coefficient in hybrids was bimodal, and individuals with a majority of the *P. sylvestris* ancestry had the smallest inbreeding values across all studied pines (Figure S7, Table S2, Table S3).

Hierarchical F‐statistics analysis showed that most genetic variation was explained by ancestry class (F_ST ancestry_ = 0.045), with negligible structure among populations within classes (F_ST population_ = −0.016). The total genetic differentiation among the four ancestry groups was moderate (0.18), consistent with mean F_ST_ values between hybrids and parental species (0.16–0.17). Pairwise F_ST_ comparisons further confirmed strong divergence between *P. sylvestris* and *P. mugo* (F_ST_ = 0.399), and relatively low differentiation within each species ( *P. sylvestris* F_ST_ = 0.019; *P. mugo* F_ST_ = 0.032), while hybrid populations exhibited greater variability (F_ST_ = 0.075) (Figure S8, Table S4). This moderate differentiation reflects the presence of two hybrid groups, with predominant *P. mugo* (H_PM) or *P. sylvestris* (H_PS) ancestry, each genetically closer to their respective parental species and more divergent from the other, consistent with directional introgression patterns (Table S4).

### 3.3 Introgression Patterns in Hybrid Zones

**FIGURE 4:**
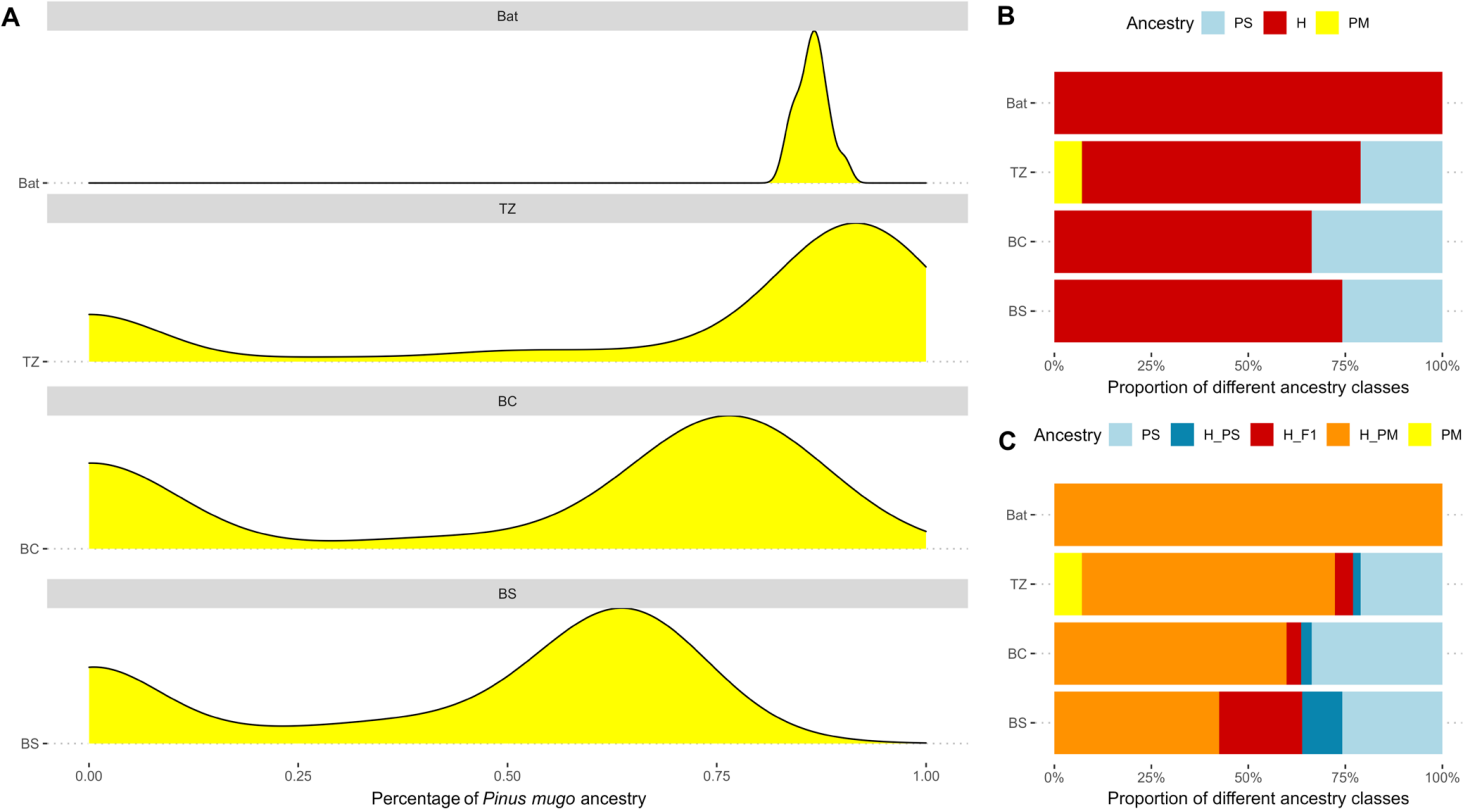
Distribution and proportions of different ancestry classes within Bat stand and each hybrid zone (TZ, BC, BS) population. (A) Density plots showing the mean proportion of *Pinus mugo* ancestry (yellow) (B) Proportions of pure *P. sylvestris* , pure *P. mugo* , and hybrids genotypes. (C) Subclasses of hybrid individuals based on their dominant ancestry: *P. mugo* ‐like hybrids (H_PM), *P. sylvestris* ‐like hybrids (H_PS), and early‐generation hybrids (H_F1). Ancestry classes are colour‐coded as in Figure 2, with the addition of dark blue for H_PS and orange for H_PM.

**FIGURE 5:**
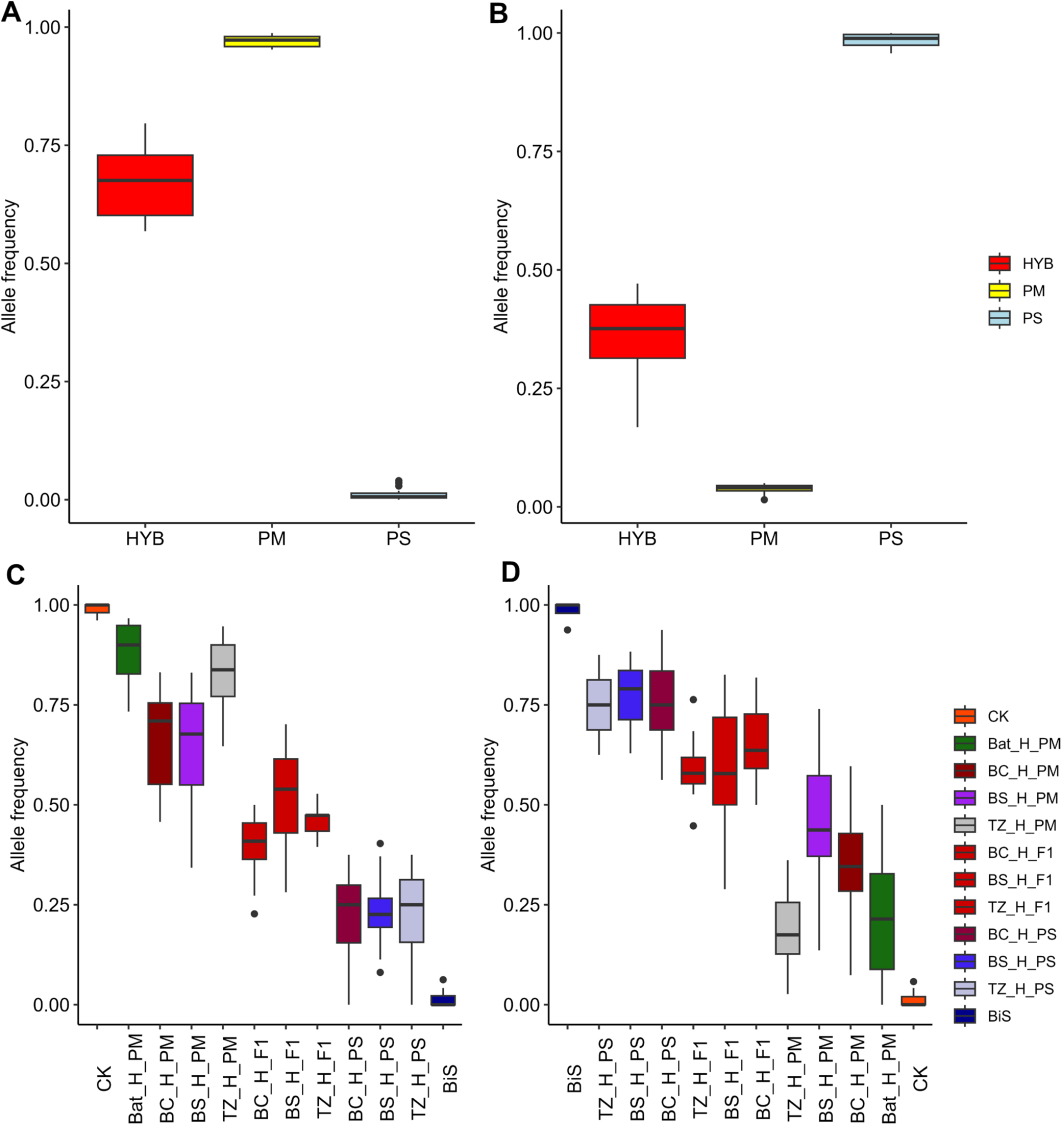
Distribution of ancestry‐informative marker (AIM) frequencies across hybrid classes. Boxplots showing allele frequency distributions of SNPs identified as ancestry‐informative markers (AIMs) for *Pinus mugo* (A) and *Pinus sylvestris* (B) across hybrid (HYB), pure PM, and pure PS classes. Boxplots showing frequency distributions of AIMs across hybrid classes and contact zones, illustrating population‐specific variation (C, D). Ancestry classes are colour‐coded as in Figure 2 and contact zones are colour‐coded as in Figure 3.

The hybrid index based on ancestry‐informative markers (AIMs) shows a strong correlation with the LEA ancestry coefficient in all SNPs (*r* ^2^ = 0.98, *p* value < 0.01), indicating that both methods are reliable for detecting hybrid ancestry (Figure S9). Analysis of the density distributions of hybrid ancestry across different hybrid zones reveals significant variation, suggesting asymmetrical introgression between *Pinus sylvestris* and *Pinus mugo* , consistent across distinct contact zones (Figure 4A). Specifically, hybrid zones such as TZ and BC exhibit a skew toward *P. mugo* ancestry, while the BS zone displays a more balanced profile, indicating regional differences in gene flow intensity. This asymmetry is further corroborated by the frequency of ancestry‐informative SNPs in hybrids. Despite high ancestral polymorphism typical of conifers, we identified 130 ancestry‐informative markers (~1.8% of 7390 SNPs) as fixed or nearly fixed between *P. sylvestris* and *P. mugo* (84 from *P. sylvestris* and 46 from *P. mugo* ). This proportion aligns with expectations under neutral coalescent models given their estimated divergence time (~6 Mya), generation time (25 years), and conifer‐specific mutation rate (~8 × 10^−10^) (Wachowiak, Palme, and Savolainen 2011; de la Torre et al. 2017). These results support the reliability of hybrid index estimates and indicate that divergence between the parental taxa is adequately captured. The mean frequency of SNPs associated with *P. mugo* was significantly higher than 0.5, while SNPs associated with *P. sylvestris* were significantly lower (0.71 vs. 0.37, respectively), highlighting a general bias toward *P. mugo* ancestry (Figure 5A,B). The unique composition of each hybrid zone is further supported by the distribution of mean AIM frequencies across different hybrid classes within each contact zone, as well as in slight variations in specific AIM frequencies (Figure 5C,D). In particular, the Bat and TZ zones exhibit the highest frequencies of *P. mugo* ‐associated SNPs, whereas the BS zone shows the highest frequencies of variants of *P. sylvestris* .

The majority of individuals found across all hybrid zones were of hybrid originand the percentage of hybrid individuals ranges from 70% to 100% of all samples (Figure 4B). Hybrid index results are further corroborated by the triangular plot analysis, which additionally shows the assignment of the majority of individuals to advanced *P. mugo* backcrosses. The results suggest the absence of one of the parental species ( *P. mugo* ) and indicate a small percentage of individuals with predominance of *P. sylvestris* ancestry (Figure 4B, Figure S10). Although some individuals based on hybrid index alone could be classified as first‐generation hybrids, they should rather be considered as later‐generation hybrids (Figure S10) based on triangular plot results. Additionally, 30 individuals from TZ identified as “pure” *P. mugo* were consistently shown in other analyses as indistinguishable from other hybrid individuals (they were clustered with hybrids on PCA and hybrid index was non‐zero), so they were treated as H_PM instead. However, the proportion of such early‐generation crosses between *P. sylvestris* and *P. mugo* is relatively low across all hybrid zones, suggesting that ongoing backcrossing and further hybridization events contribute to the formation of later‐generation hybrids with mixed ancestry (Figure 4C).

This unidirectional pattern of introgression is further supported by chloroplast DNA (*cp*DNA) analysis. Most F1 hybrids exhibit *P. mugo cp*DNA, while *P. sylvestris cp*DNA is primarily restricted to backcross hybrids and individuals with higher *P. sylvestris* ancestry (Figure S11). This unidirectional chloroplast inheritance suggests that *P. mugo* typically serves as the paternal parent in hybridization events, implying a bias in pollen dispersal or hybrid establishment favoring also *P. mugo* as the maternal lineage. The consistency of *P. mugo cp*DNA between hybrid zones and hybrid types indicates that this pattern is not random but reflects a systematic bias in the direction of gene flow.

**FIGURE 6:**
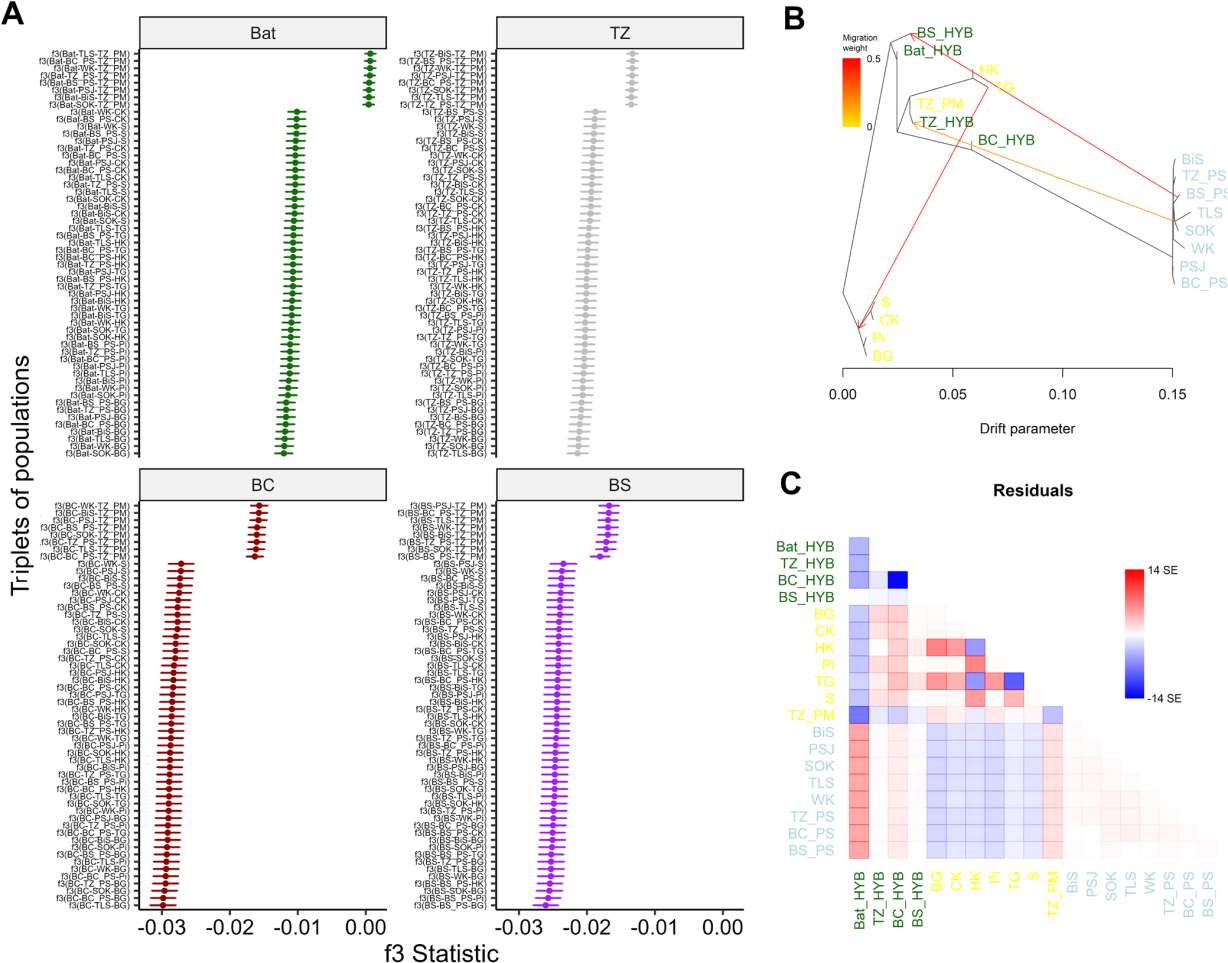
Inference of admixture and historical gene flow among hybrid pine populations. (A) Results of *f*_3_‐statistics for Bat stand and hybrid populations (Bat, TZ, BC, BS). Each row shows tests of the form *f*_3_(X; A, B), where X is the target population and A/B are source populations. Significantly negative values (with standard errors) indicate a signal of admixture. (B) TreeMix maximum‐likelihood tree showing the optimal number of migration edges inferred among populations. Arrows represent gene flow direction and weight, with warmer colours indicating stronger migration signals. (C) Residual covariance matrix from TreeMix, highlighting population pairs with excess (blue) or deficit (red) of shared genetic drift compared to the tree‐only model. Colour intensity corresponds to standard error deviation from expectation.

The results of the f3 test provide strong evidence of admixture in our pine populations, as indicated by significantly negative f3 values across all triplets tested (Figure 6A, Table S5). Interestingly, two populations of *P. mugo* (BG, Pi) were consistently associated with the triplets that exhibited the most negative f3 statistics in all hybrid zones. The f3 test also corroborates earlier findings regarding the 30 individuals from TZ identified as “pure” *P. mugo* . The consistent occurrence of more positive f3 values in all hybrid zones for triplets including TZ_PM as the source population of *P. mugo* suggests that these individuals should instead be considered hybrids. The least negative f3 values were observed in the *P. uliginosa* population (Bat), which could suggest the presence of unrecognised source populations or may reflect a stronger genetic drift within this population as it can obscure evidence of admixture in the f3 test.

TreeMix analysis revealed a consensus maximum likelihood tree with three optimal migration events, collectively explaining 98.1% of the variation in the data (Figure 6B, Figure S12). The genetic relationships depicted in this tree align with patterns observed in the PCA and LEA analyses, indicating significant divergence between the parental taxa, *P. mugo* and *P. sylvestris* , while illustrating a clear pattern of directional gene flow biased toward *P. mugo* . A prominent signal of gene flow was identified between *P. sylvestris* and the BS contact zone and reference *P. uliginosa* stand (Bat), while another migration edge linked *P. sylvestris* with the branch containing the TZ and BC populations. The drift parameters, as inferred from the branch lengths, suggest a strong genetic divergence between dwarf mountain pines from the Tatra Mountains (HK, TG) and the rest of the *P. mugo* populations, with hybrid populations exhibiting closer genetic similarity to the Tatra populations. Interestingly, substantial gene flow was also detected between these two groups of dwarf mountain pines. Among hybrid populations, the drift parameters reflect geographic proximity: populations close to each other (BS and Bat) display the shortest branch lengths, followed by TZ in the Sudetes Mountains. In contrast, the BC contact zone, located in the foothills of the Nowotarska Valley in the Tatra Mountain, exhibits the longest branch lengths and the greatest genetic divergence. The residuals of the models (Figure 6C) highlighted significant deviations from the drift‐only model, with positive residuals particularly pronounced in populations exhibiting extensive hybridization, such as BC.

### 3.4 Variability of Phenotypes Within the Hybrid Zone

**FIGURE 7:**
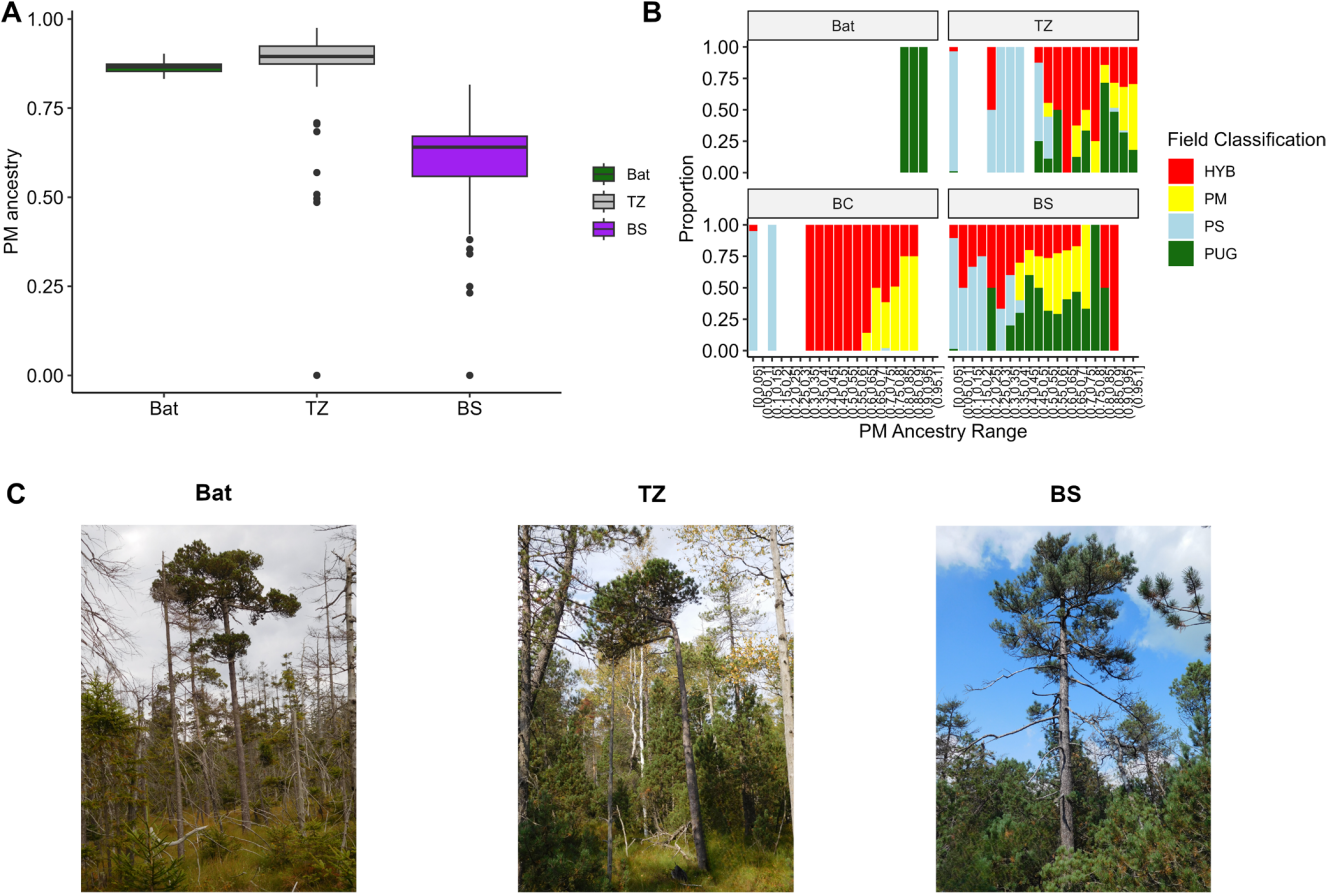
Relationship between genetic ancestry and field‐assigned morphological classifications in hybrid zones. (A) Boxplots of *Pinus mugo* (PM) ancestry proportions of individuals classified according to morphology as *P. uliginosa* in three analysed stands (Bat, TZ, BS). Notice, that there were no individuals of this phenotype in BC contact zone. (B) Stacked barplots displaying the distribution of field‐based morphological classifications: *P. sylvestris* (PS), *P. mugo* (PM), *P. uliginosa* (PUG), and hybrids (HYB),across PM ancestry ranges within each population. Field‐classified *P. uliginosa* individuals consistently show high PM ancestry, though similar genetic profiles also correspond to a wide range of other phenotypes. (C) Representative phenotypes of individuals from each hybrid zone identified as *P. uliginosa* during sampling.

The distribution of phenotype classes across the hybrid zones (HZ) exhibits a complex relationship between ancestry and morphology, particularly within individuals with a high proportion of *P. mugo* ancestry. Hybrid pines display a broad spectrum of phenotypic traits that do not always align predictably with their genetic background, making field identification challenging. Specifically, it was anticipated that increased *P. mugo* ancestry would correlate with more shrub‐like forms characteristic of classic *P. mugo* , yet this pattern does not consistently hold (Supporting Information S2). This inconsistency is further exemplified by the occurrence of individuals classified as *P. uliginosa* , a phenotype considered a distinct species due to its unique morphological traits and described in Wielkie Torfowisko Batorowskie (Bat). In this population, all individuals were identified as *P. uliginosa;* there were no other pines growing there, and they all display a significant *P. mugo* ancestry, averaging nearly 85%; yet these individuals exhibit a tall tree‐like form atypical of shrub‐dominated *P. mugo* (Figure 7). Individuals of the same phenotype were also observed and included in our sampling in TZ and BS, but they were absent in BC. However, although field‐classified *P. uliginosa* consistently shares a similar genetic ancestry profile, the same proportion of ancestry could produce a variety of other phenotypes (Figure 7). This phenomenon suggests that a similar genetic background, resulting from the crossing of parental species, can yield markedly different morphological outcomes, likely influenced by environmental factors or complex genetic interactions.

## 4 Discussion

### 4.1 Environmental Constraints on Hybrid Zone Formation

Our study identifies a consistent pattern of hybrid formation across all investigated contact zones between *Pinus sylvestris* and *P. mugo* , each dominated by admixed individuals. This suggests that hybridization between these two species is not random, but rather a predictable outcome occurring wherever suitable ecological niches permit hybrid persistence. Mountain and foothill peatlands represent such niches, with their formation closely linked to climatic and ecological changes following the Last Glacial Maximum (LGM). Notably, all currently recognized natural hybrid zones between these species occur in proximity to mountain ranges (Christensen 2008; Klobucnik et al. 2022; Kormutak et al. 2017). This distribution, coupled with the present‐day altitudinal separation of the parental species, supports the hypothesis that hybridization events were facilitated primarily by post‐glacial shifts in species distributions and were spatially limited (Szczepański et al. 2024).

Palynological data, including pollen analyses from sediment cores collected from the investigated hybrid zones (Gajewski et al. 2001; Binney et al. 2017), indicate increased peatland formation approximately 14 kya, peaking around 9 kya, thus providing an upper temporal boundary for the establishment of these hybrid populations (Boratyński 1978; Madeyska 2005; Łajczak 2009). During the LGM, non‐glaciated regions of Europe, especially areas adjacent to mountain ranges, were dominated by treeless Arctic scrub and herbaceous vegetation, offering low‐elevation refugia for cold‐adapted species like *P. mugo* (Svenning et al. 2008; Janská et al. 2017). Palaeoecological reconstructions suggest that *P. sylvestris* began recolonizing Central Europe between 14 kya and 12.5 kya, reaching its full distribution around 8 kya (Cheddadi 2006). As peatlands expanded in the post‐glacial landscape, novel ecological niches emerged, enabling secondary contact between northward‐migrating *P. sylvestris* and resident *P. mugo* populations. Consequently, persistent hybrid zones were established, as evidenced by pine fossils identified in peat‐bog cores at the early stage of their formation documenting Holocene timberline fluctuations in the mid‐mountains of Central Europe (Treml et al. 2006). Furthermore, their ancient origin is evident from the highly admixed genomic backgrounds and varying proportions of parental ancestry observed in the present‐day hybrid populations. Thus, despite the inherent longevity and extended generation times characteristic of conifers (Wachowiak, Salmela, et al. 2011; Lanner 2002), the relatively brief period (only a few thousand years) of sympatric occurrence has been sufficient for these areas to develop into stable hybrid zones dominated by genetically admixed individuals characterized by various proportions of parental species ancestry.

### 4.2 Asymmetric Introgression Across Hybrid Zones

Although environmental factors primarily shaped the initial formation of hybrid zones, the persistence and genomic composition of these zones depend significantly on demographic factors, genetic variation, reproductive biology, and selective pressures acting on hybrids (Kawakami and Butlin 2012; Barton and Hewitt 1985; Arnold 1997). Our data indicate that hybrid individuals constitute 70%–100% of the sampled populations in all studied hybrid zones. However, hybrid classes and parental contributions to these admixed populations vary notably in their proportions. A consistent feature across the studied contact zones is asymmetric, predominantly *P. mugo* ‐biased introgression, with hybrids displaying significantly higher ancestry proportions derived from *P. mugo* . An exception was noted at the Błędne Skały hybrid zone, where we observed a slight increase in *P. sylvestris* ancestry, though it still remained secondary to *P. mugo* .

Demographic processes, such as differences in effective population sizes of the parental species, may partially explain the repeated pattern of asymmetric introgression. Typically, genetic contributions from less abundant parental species decline over time in hybrid zones (Penalba et al. 2024). It is plausible that during initial secondary contact, northward‐expanding populations of *P. sylvestris* were initially smaller relative to locally abundant *P. mugo* , favouring asymmetric backcrossing toward *P. mugo* (Excoffier et al. 2009). However, demography alone cannot fully account for the continued bias toward *P. mugo* ancestry. For instance, despite the subsequent retreat of *P. mugo* to higher elevations and a simultaneous expansion of *P. sylvestris* populations, the introgression pattern remains strongly skewed toward *P. mugo* . Additionally, at Bór na Czerwonem, located adjacent to dense stands of *P. sylvestris* , we did not detect any significant increase in *P. sylvestris* ancestry among hybrids and we did not observe early generation hybrids with *P. sylvestris* chloroplast genome, further emphasising a persistent directional bias in gene flow toward *P. mugo* .

Alternative mechanisms, including intrinsic prezygotic barriers such as phenological mismatches or gametic incompatibilities, as well as postzygotic processes like early‐stage hybrid inviability, could also result in directional hybridization. On lowland sites, Scots pine typically releases pollen earlier than *P. mugo* , increasing the likelihood that *P. mugo* is pollinated by *P. sylvestris* , as pine cones are receptive before the species releases its own pollen (Boratyński et al. 2003). While mountain environments and specific climatic conditions on the peat bogs may alter this general phenological pattern, unfortunately, no detailed phenological observations are currently available from the hybrid zones analyzed in this study. However, indirect evidence from our analyses suggests that phenological differences are unlikely to be the primary drivers of asymmetric gene flow. For example, the geographically proximate hybrid zones at Błędne Skały (BS) and Torfowisko pod Zieleńcem (TZ), separated by only ~30 km, exhibit marked differences in parental ancestry proportions, which phenological shifts alone cannot easily explain. Furthermore, controlled experimental crosses between *P. sylvestris* and *P. mugo* have produced mixed outcomes, either showing no obvious barriers (Kormutak et al. 2017) or revealing a directional bias favoring *P. mugo* as the paternal tree (Wachowiak, Celiński, and Prus‐Głowacki 2005). Notably, our findings show that the majority of early‐generation hybrids carried chloroplast DNA from *P. mugo* , indicating consistently paternal inheritance from *P. mugo* in natural hybridization events. Similarly, all observed backcrosses to *P. mugo* retained *P. mugo* chloroplast DNA, reinforcing the view that while initial hybridization events may occur bidirectionally, subsequent selection appears to favor progeny from crosses with *P. mugo* as the pollen donor. The cytonuclear incompatibility between the *P. sylvestris* chlorotype and *P. mugo* nuclear/mitochondrial genomes cannot be ruled out to support a pattern of biased *P. mugo* ancestry of most hybrids. Such incompatibilities have been suggested in controlled crossing experiments between these species (Kormutak et al. 2017), though further replication and more detailed experimental work are needed to confirm this mechanism. It seems that selection acts during early development, possibly at fertilization, the seedling, or juvenile stage, depending on specific parental genomic background.

Such selection‐driven filtering of hybrid individuals may facilitate adaptive introgression, where beneficial alleles from parental species are preferentially retained in hybrids, enhancing their fitness in specific ecological conditions and contributing to the long‐term persistence of the hybrid zone (Arnold 1997; Penalba et al. 2024; Curry 2015). The transfer of environment‐specific advantageous alleles from one species to the other in the hybrid zone has been well documented in herbaceous plant genera such as *Senecio* and *Helianthus* (Whitney et al. 2010; Whitney et al. 2006; Kim et al. 2008), as well as in long‐lived tree species like spruce, eucalyptus, oak, and poplars, where interspecific gene flow has contributed to increased environmental tolerance and enhanced fitness in specific habitats (Hamilton et al. 2014; de Lafontaine et al. 2015; Larcombe et al. 2015; Abadie et al. 2012; Suarez‐Gonzalez et al. 2016; Suarez‐Gonzalez et al. 2018). Additionally, primarily ecological or habitat‐related pressures may be influencing site‐specific variations in hybrid ancestry. Indeed, the hybrid zone at Błędne Skały is the only site where hybrids occupy sandstone rock formations with a thin layer of soil and show a slight shift toward *P. sylvestris* ancestry. Such a pattern suggests a major role of selection in shaping the hybrid composition in this area as compared to the other two analyzed peatland populations.

### 4.3 Elevated Genetic and Phenotypic Variation Across Hybrid Zones

The existence of some early generations of hybrid trees reported here and hybrid seeds reported in some earlier studies (Wachowiak, Celiński, and Prus‐Głowacki 2005; Kormutak et al. 2019; Wachowiak, Lewandowski, and Prus‐Głowacki 2005) indicates ongoing interspecific gene flow. The results suggest that the analysed populations reflect continued hybridization processes that have unfolded over millennia. The slightly different ancestry proportions by site seem unlikely to result from old hybridization between the species followed by expansion of hybrid genotypes to their present‐day occurrence. The observed variations in hybrid ancestry correlate with environmental factors and show that the proportion of hybrids with *P. sylvestris* ancestry (H_PS) is very similar at both BC and TZ ‘wet’ peatland stands but a few times lower as compared to ‘dry’ BS sandstone. At the BS site, we also detected an excess of early‐generation hybrids and individuals with a predominantly *P. sylvestris* genomic signature. This observation, together with the predominance of admixed individuals over pure parental forms across the contact zones studied, supports the bounded hybrid superiority model for hybrid zone maintenance. Under this model, hybrids or introgressed individuals experience environment‐dependent selection in novel habitats and can outperform parental taxa (Arnold 1997; Moore 1977). Enhanced recombination and transgressive segregation within hybrid populations generate elevated genetic and phenotypic variation relative to parental species. Consistent with this prediction, our analyses revealed a great diversity of phenotypic forms, significantly higher genetic diversity and lower inbreeding coefficients among hybrids compared to parental populations, with early‐generation (putative F1) hybrids exhibiting the greatest genetic variation. This genetic enrichment aligns with patterns observed in other plant hybrid zones, frequently described as “melting pots” of genetic diversity (Arnold 1997). Similar observations across diverse taxa indicate that hybrid zones are critical reservoirs for novel allelic combinations, potentially facilitating adaptive responses to environmental changes (Moran et al. 2021; Penalba et al. 2024; Rieseberg et al. 2007; Rieseberg and Willis 2007; Abbott et al. 2013; Nieto Feliner et al. 2023).

Transgressive phenotypes arising from increased genetic variation offer immediate targets for selection, sometimes conferring adaptive advantages in specific ecological niches. Hybrid pines exhibited considerable phenotypic diversity across hybrid zones, and phenotypes were not always strongly predicted by genomic ancestry. For instance, individuals with as little as 10% *P. sylvestris* ancestry occasionally displayed growth forms characteristic of this parental species. We documented a remarkable diversity of growth forms, including small ground‐covering shrubs, multi‐stemmed shrubs (3–5 m in height), and trees with unique crown architectures or pronounced stem curvatures. Field observations further revealed a nonrandom spatial distribution of these phenotypes within hybrid zones: *P. mugo* ‐like phenotypes predominated in the wettest parts of peatland, whereas *P. sylvestris* ‐like individuals were typically found at the outskirts or in drier areas. This pattern implies that water availability gradients within hybrid zones likely exert differential selective pressures on transgressive phenotypes, favouring forms best adapted to specific microhabitats.

Moreover, while similar selective pressures may explain convergent phenotypes and genomic compositions observed in peatland‐associated hybrid zones (such as Torfowisko pod Zieleńcem and Bór na Czerwonem), distinct environmental selection pressures have likely shaped the unique genomic and phenotypic composition observed in the Błędne Skały hybrid zone established on dryer, thin‐soiled sandstone outcrops. This underscores how hybridization between the same parental species can lead to similar yet distinctly adapted hybrid outcomes, depending on local ecological conditions. Such environmentally driven differentiation among hybrid zones parallels the case of hybrid sunflowers ( *Helianthus anomalus* , *H. deserticola* , and *H. paradoxus* ), which originated from the same parental cross but occupy distinct niches (Rieseberg et al. 2003; Rieseberg et al. 2007), as well as the hybrid spruce *Picea brachytyla*, where two parental taxa gave rise to more than one homoploid hybrid species adapted to different ecological contexts (Wang et al. 2023). However, evidence for multiple homoploid hybrids arising from identical parental crosses remains relatively limited. Given these complexities, future studies should focus on verifying selective pressures governing hybrid persistence, assessing fitness consequences of introgressed alleles, and identifying genomic signatures of adaptive introgression to comprehensively understand the long‐term evolutionary consequences of hybridization in pines.

### 4.4 Hybridization as a Pathway to Speciation

Our results indicate that the hybrid pine populations studied here represent distinct points along a speciation continuum, characterised by relatively recent population (~10,000 years) of stabilised hybrids predominantly comprising introgressed *P. mugo* individuals. These populations have been shaped by strong selective pressures in habitats unsuitable for pure parental species. We provide robust genomic evidence supporting the conclusion that the peat bog pine, *P. uliginosa* , is a partially stabilised hybrid lineage, shaped by ecological filtering and demographic isolation but lacking sufficient genetic divergence to be recognised unequivocally as a distinct species. Therefore, *P. uliginosa* is best considered as a hybrid ecotype with evolutionary and conservation significance.

Initially, *P. uliginosa* was recognised as a distinct species primarily due to its restricted distribution, distinctive morphology, and highly specialised habitat requirements. Intriguingly, this taxon was first described from Stołowe National Park at Wielkie Torfowisko Batorowskie, the only known locality where it occurs allopatrically, without the sympatric presence of either parental species, *P. sylvestris* or *P. mugo* (Woronko 2002; Danielewicz and Zieliński 2000; Marek 1988). Subsequently, similar morphological forms observed in other peat bogs across Poland, Slovakia, and the Alps were also classified as *P. uliginosa* . However, these additional populations never occur in isolation; rather, they coexist with parental species and numerous intermediate hybrid phenotypes, complicating taxonomic assignments (Christensen 2008; Businský and Kirschner 2010; Christensen and Dar 2008). Our genomic clustering analyses challenge the concept of *P. uliginosa* as a distinct species, instead placing it firmly within a continuous hybrid spectrum between *P. mugo* and *P. sylvestris* . Thus, our data support a simpler taxonomic scenario involving only two parental species and their hybrids. Notably, individuals identified as *P. uliginosa* at the locus classicus (Wielkie Torfowisko Batorowskie) consistently showed ~85% genomic ancestry from *P. mugo* . Despite this predominant genomic ancestry from shrubby dwarf pine, these hybrids exhibit mostly a single‐stem phenotype of unexpectedly tall growth form up to 20 m.

Interestingly, a similar phenotype has been observed in other hybrid zones, yet phenotype alone does not always align with genomic ancestry proportions. Indeed, individuals with comparable genomic backgrounds frequently display diverse growth forms (Supporting Information S2). Moreover, in the hybrid zone at Bór na Czerwonem in the foothills of the Tatra Mountains, hybrids with ancestry proportions similar to those at Wielkie Torfowisko Batorowskie did not develop the characteristic *P. uliginosa* morphology. This underscores that the *P. uliginosa* ‐like phenotype is not a fixed endpoint of hybrid evolution between *P. mugo* and *P. sylvestris* , but rather a potential outcome contingent upon the particular evolutionary trajectory and local genotype‐environmental interaction at each hybridization site.

While identifying exact source populations involved in hybridization is challenging due to the weak phylogeographic structure of both parental species, our genomic data offer important insights. Notably, we found that *P. mugo* populations contributing to hybrids in the Stołowe Mountains likely originated not from the geographically proximate Karkonosze Mountains but from more distant mountain ranges, such as the Tatra and Beskid Mountains. Although the Karkonosze Mountains served as a refugium for various taxa during the Last Glacial Maximum (LGM) (Engel et al. 2010), this relict population does not appear to have significantly contributed to the hybridization events observed. Instead, our data indicate that ancestral *P. mugo* populations were historically more widespread, thus representing the likely source population involved in hybrid formation in the Stołowe Mountains. This interpretation is further supported by the distinct haplotypic patterns of the Karkonosze population compared to neighbouring hybrid zones, as previously documented (Szczepański et al. 2024).

### 4.5 Taxonomic and Conservation Implications

As demonstrated in our study, sympatric occurrence of *Pinus sylvestris* and *P. mugo* results in a diverse array of hybrid phenotypes and genotypes, complicating individual taxonomic assignments. Hybrids between these two species have generally been classified as *Pinus ×rhaetica* Brūgger (Brügger 1886); however, phenotypically distinct hybrids have been assigned various taxonomic names within the broader *Pinus mugo* complex across different geographic locations (Christensen 2008; Businský and Kirschner 2010). Some of these forms, such as *P. uliginosa* , have been included in national Red Lists and afforded legal protection (Mirek 2006), primarily due to distinctive phenotypes and limited distribution, i.e., factors that historically led to their misclassification as separate species. Based on our genetic findings, we propose that individuals resembling *P. uliginosa* , along with other hybrid forms described in various *P. sylvestris* – *P. mugo* contact zones, should consistently be classified as *Pinus* × *rhaetica* or more generally as *Pinus mugo* × *Pinus sylvestris* . Molecular genetic assessments, such as those presented here, provide essential evidence to clarify hybrid identities and streamline taxonomy within the *Pinus mugo* complex.

Regardless of current taxonomic status, the long‐term persistence of these hybrid populations appears uncertain, as they generally represent transient evolutionary outcomes rather than stable species. Their selective advantages over parental species in specific habitats, particularly peatlands, are paradoxically linked to their vulnerability, given current climatic trends such as habitat drying and warming‐induced environmental shifts (Holt 1990; Weltzin et al. 2003; Turetsky et al. 2015). Furthermore, no evidence indicates that these hybrids can survive or persist beyond their specialised habitats (Szczepański et al. 2024). Consequently, these lineages face rapid genomic erosion driven by habitat fragmentation and desiccation, diminishing their adaptive potential and increasing susceptibility to environmental stress. Thus, preserving the genomic diversity contained within these hybrid populations should be prioritised. Yet, assigning conservation status may prove challenging since existing regulatory frameworks often overlook hybrids as distinct management entities, creating ambiguity in conservation policies. This issue is compounded by the fact that neither parent species, *P. sylvestris* nor *P. mugo* , is currently considered endangered. Consequently, hybrid populations risk neglect from habitat‐centric conservation strategies that inadvertently prioritise parental or other non‐hybrid species (Todesco et al. 2016; Hamilton and Miller 2016). Additionally, wetland restoration or other habitat‐focused management actions may inadvertently disrupt hybrid zones by altering ecological conditions, leading to further loss of hybrid diversity.

This situation highlights broader conservation dilemmas surrounding hybridization specifically, when hybridization should be actively managed or when it should be recognised as a critical evolutionary process. Historically, hybridization between natural stands of *P. sylvestris* and *P. mugo* has occurred spontaneously in mountain regions, whereas anthropogenic introductions of *P. mugo* outside its native range have also led to hybridization events (Danusevičius et al. 2012). Therefore, while hybridization resulting from non‐native species introductions may justify active management to protect genetic integrity, naturally occurring hybridization should generally be permitted to continue without intervention (Grabenstein and Taylor 2018). Indeed, as shown in our research, these natural hybrid zones represent invaluable evolutionary laboratories where genomic innovations emerge and incipient speciation processes unfold. Protecting these unique hybrid stands is therefore crucial, as they constitute biological heritage areas that provide fundamental insights into biodiversity formation.

## Author Contributions

**Bartosz Łabiszak:** conceptualization, data curation, formal analysis, methodology, software, visualisation, writing – original draft, writing – review and editing. **Sebastian Szczepański:** writing – original draft, writing – review and editing. **Witold Wachowiak:** conceptualization, investigation, funding acquisition, project administration, resources; supervision; validation, writing – original draft, writing – review and editing.

## Benefits Generated

Benefits from this research accrue from the sharing of our data and results on public databases as described above.

## Conflicts of Interest

The authors declare no conflicts of interest.

## Supporting information


**Data S1:** mec70137‐sup‐0001‐Data_S1.csv.

**Data S2:** mec70137‐sup‐0002‐Data_S2.pdf.

**Figure S1:** Geographic ranges of pine species and locations of studied populations.
**Figure S2:** Distribution of transcriptome contig mapping across retained SNPs.
**Figure S3:** Cross‐entropy criterion from LEA analyses. Cross‐entropy values across 10 replicate runs for each *K* (number of clusters), ranging from 1 – 10. The optimal number of clusters is indicated by the first substantial decrease in cross‐entropy at *K* = 2.
**Figure S4:** Population structure of parental species. Principal component analysis (PCA) of *P. mugo* and *P. sylvestris* individuals from allopatric populations. Individuals are colour‐coded by population as in Figure 2.
**Figure S5:** Population structure within each hybrid zone. Principal component analysis (PCA) of individuals projected along PC1 and PC2, shown separately for each hybrid zone. Reference allopatric populations of both parental species are included for comparison. Individual trees are consistently colour‐coded by their population of origin. Allopatric populations of *P. mugo* and *P. sylvestris* are represented using gradients of their respective primary colours (as in Figure 1), while contact zone populations are shown in distinct colours.
**Figure S6:** Genetic diversity levels in studied pines. Boxplots comparing mean values of observed heterozygosity, expected heterozygosity, allelic richness, and fixation index (*F*‐index) among individuals grouped by ancestry class: putative F1 (F1), later generation hybrids (H), pure *Pinus mugo* (PM), and pure *Pinus sylvestris* (PS).
**Figure S7:** Genetic diversity metrics by population and ancestry class. Barplots showing observed heterozygosity, expected heterozygosity, allelic richness, and fixation index (F‐index) for individuals grouped by population of origin and ancestry class. Ancestry classes are colour‐coded as in Figure 3, with the addition of dark red to represent putative F1 hybrids.
**Figure S8:** Pairwise F_ST_ differentiation between populations grouped by ancestry class. Heatmap showing pairwise F_ST_ values representing genetic differentiation among populations. Populations are ordered and grouped according to their genetic ancestry class and geographic origin. Colour intensity indicates the degree of genetic divergence, with darker shades representing higher F_ST_ values.
**Figure S9:**. Correlation between hybrid index and genomic ancestry coefficient. Scatterplot showing the strong positive correlation between individual hybrid index values and genomic ancestry coefficients. The red regression line indicates a robust linear relationship (*R* ^*2*^ = 0.98, *p* < 2.2 × 10^−16^), demonstrating high concordance between the two measures of hybridization and ancestry.
**Figure S10:** Hybrid index and interclass heterozygosity across populations.
Top panel: Hybrid index values for individuals across populations, estimated using the *gghybrid* package based on ancestry‐informative markers (AIMs). Individuals are colour‐coded by population. Dashed lines indicate thresholds for hybrid class inference. Bottom panel: Triangular plot of hybrid index versus interclass heterozygosity, generated using the *triangular* R package. This plot visualises the distribution of individuals across hybrid classes and degrees of admixture, with colour‐coding matching population assignments in the top panel. Population colours correspond to those in Figure 2.
**Figure S11:** Nuclear and cytoplasmic ancestry patterns across hybrid populations.
**Figure S12:** Determination of the optimal number of migration edges in TreeMix analysis using an Evanno‐like method. Top: Mean log‐likelihood values (± SD) across 500 TreeMix replicates for each number of migration edges (*m*, from 0 to 7), shown with the proportion of variance in the data explained (red points). The horizontal dashed line indicates the 99.8% variance threshold. Bottom: Second‐order rate of change in log‐likelihood (Δ*m*), calculated following an Evanno‐like approach using the OptM R package. A significant drop at *m* = 3 indicates the most likely number of migration edges that best balances model fit and complexity.
**Table S1:** Geographic location and sample size of investigated populations. List of hybrid and allopatric populations included in the study, with corresponding acronyms, sample sizes (*N*), population names, and geographic coordinates (longitude and latitude in WGS84). All listed individuals were used in subsequent genetic and morphological analyses.
**Table S2:** ANOVA results for genetic diversity statistics among parental and hybrid groups. Analysis of variance (ANOVA) results for allelic richness (Ar), fixation index (Fi), expected heterozygosity (He), and observed heterozygosity (Ho), comparing: (Left) three population groups— *Pinus mugo* (PM), *P. sylvestris* (PS), and hybrids (H); (Right) four groups—PM, PS, hybrids (H), and early‐generation hybrids (H_F1). Significance levels: *p* ≤ 0.05 (*), *p* ≤ 0.01 (**).
**Table S3:** Tukey HSD post hoc pairwise comparisons for genetic diversity statistics. Results of Tukey's Honestly Significant Difference (HSD) tests for allelic richness (Ar), fixation index (Fi), expected heterozygosity (He), and observed heterozygosity (Ho), following ANOVA across:(1) three population groups— *Pinus mugo* (PM), *P. sylvestris* (PS), and hybrids (H); and (2) four groups—PM, PS, hybrids (H), and early‐generation hybrids (H_F1). Reported values include mean differences between groups, 95% confidence intervals, adjusted *p*‐values, and significance levels. Significance codes: *p* ≤ 0.05 (*), *p* ≤ 0.01 (**), ns = not significant. Non‐significant comparisons are additionally shaded for clarity.
**Table S4:** Mean pairwise F_ST_ values within and among hybrid and parental groups. Values represent mean Weir and Cockerham's F_ST_ estimates between genetic groups: PM— *Pinus mugo* ; PS— *Pinus sylvestris* HYB—all hybrid individuals; H_PM—hybrids with majority PM ancestry; H_PS—hybrids with majority PS ancestry. These values summarise genetic differentiation within and among parental and hybrid ancestry groups.
**Table S5:** Results of *f*₃‐statistic tests for admixture among population trios. Each row presents the outcome of a three‐population test in the form *f*₃(X; A, B), where X is the target population and A and B are putative source populations. The table reports the *f*₃‐statistic estimate (Est), Z‐score (Z), and corresponding *p* value (P). Significantly negative *f*₃ values (Z < −3) provide evidence of historical admixture in the target population.

## Data Availability

The datasets generated and analysed during the current study are available in the DRYAD repository at https://doi.org/10.5061/dryad.xwdbrv1rg.
