# Supplementary material for: Repeatable Genomic Outcomes Along the Speciation Continuum: Insights From Pine Hybrid Zones (Genus Pinus)
Source: Mol Ecol. 2025 Oct 13;34(22):e70137. doi: 10.1111/mec.70137 (PMC12617033; doi:10.1111/mec.70137)

MOLECULAR ECOLOGY

Supplemental Information S2 for:

Repeatable genomic outcomes along the speciation continuum: insights from pine hybrid zones (genus *Pinus*)

Łabiszak Bartosz, Szczepański Sebastian, Wachowiak Witold

MOLECULAR ECOLOGY

P. mugo ancestry 100%

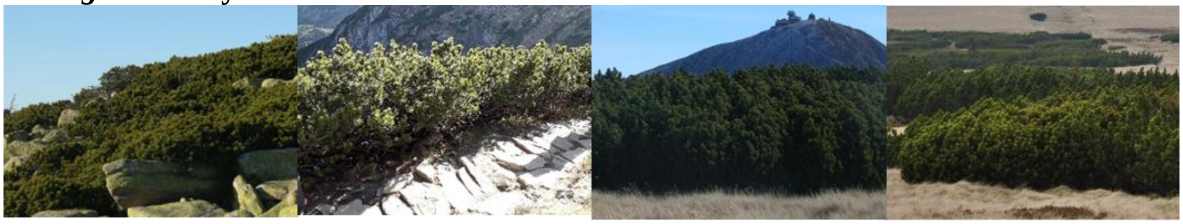

P mugo ancestry ~90%

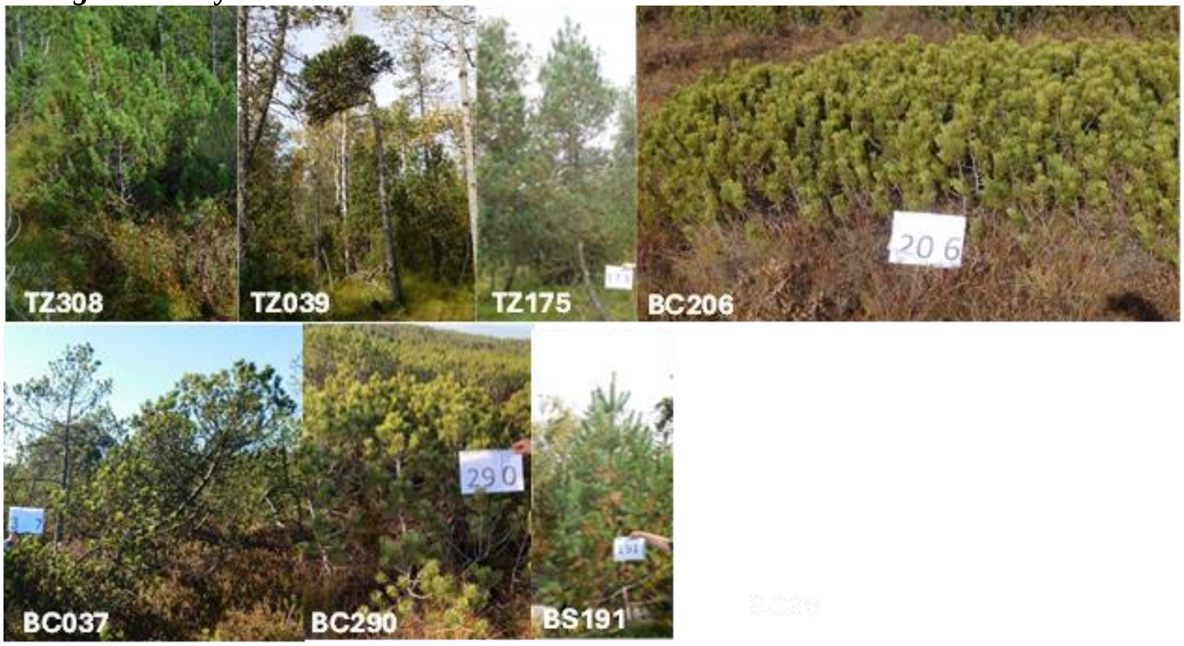

P mugo ancestry ~80%

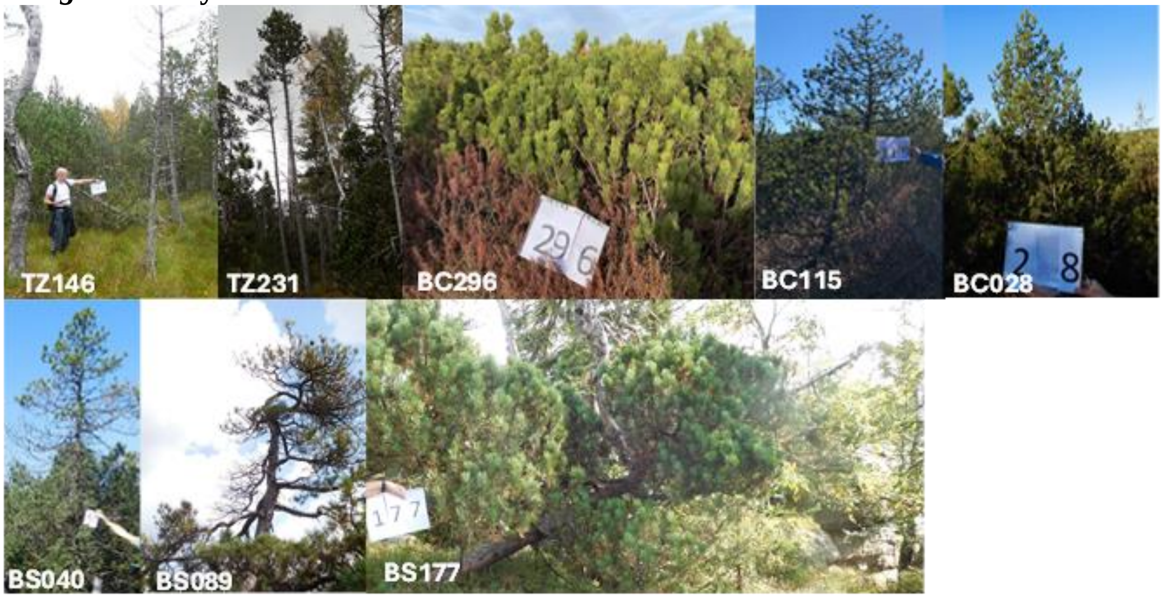

MOLECULAR ECOLOGY

***P mugo* ancestry ~70%**

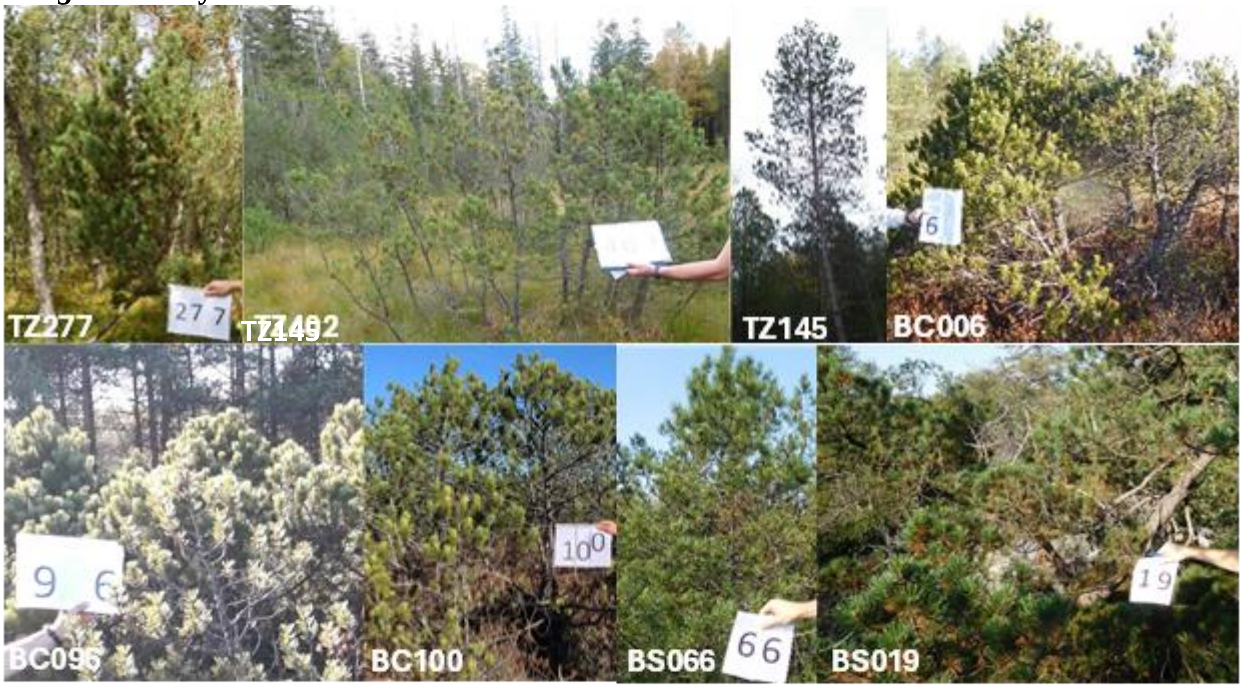

***P mugo* ancestry ~60%**

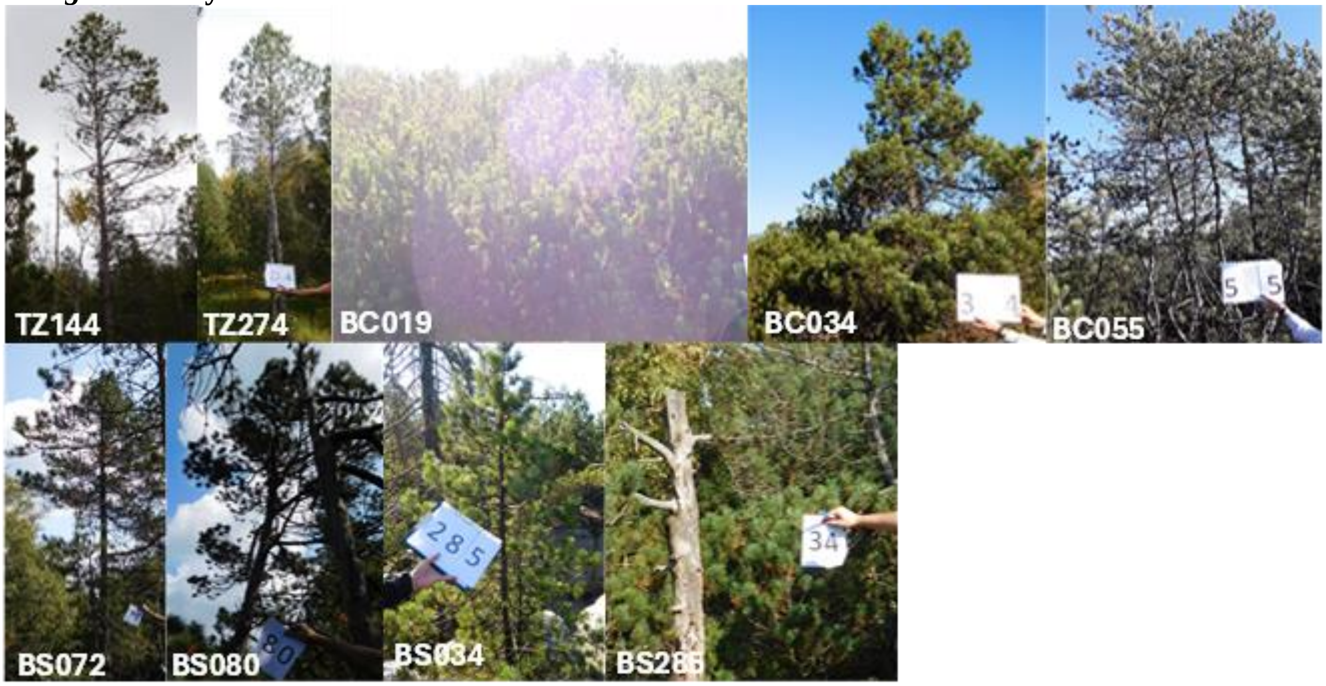

MOLECULAR ECOLOGY

P mugo **ancestry ~50 %**

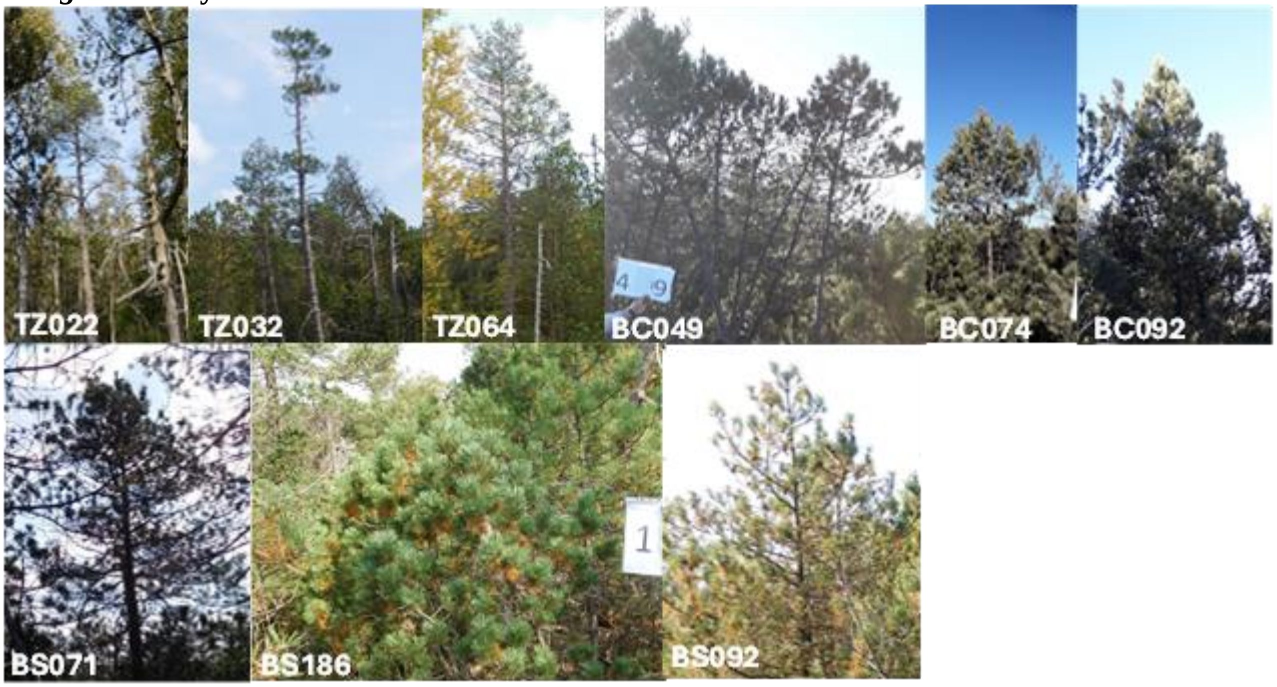

P mugo **ancestry ~40 %**

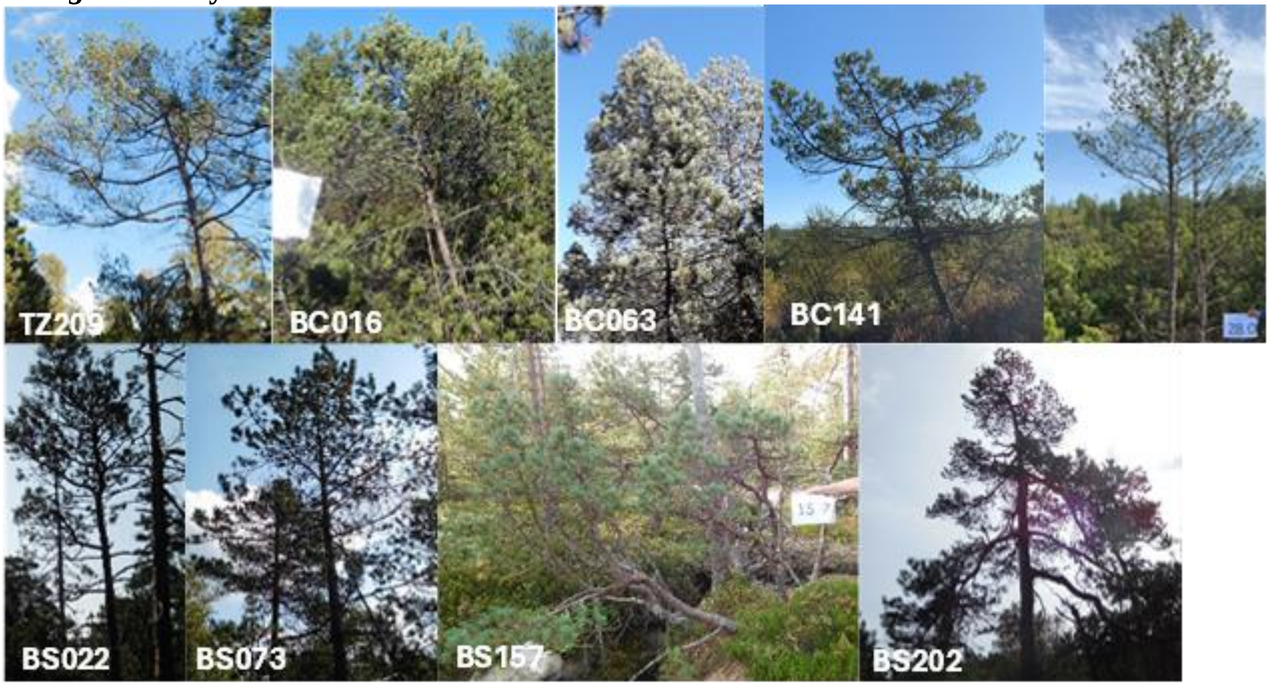

MOLECULAR ECOLOGY

P mugo **ancestry ~30 %**

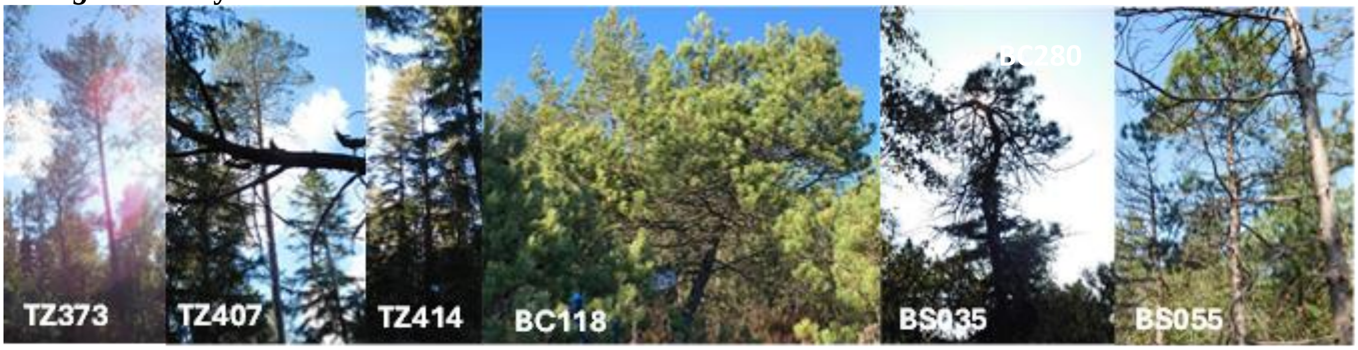

P mugo **ancestry ~20 %**

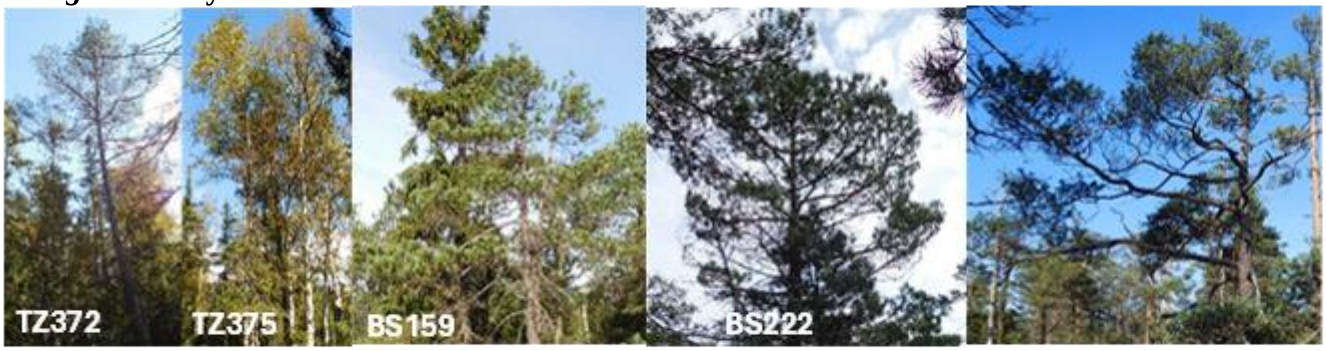

P mugo **ancestry ~10 %**

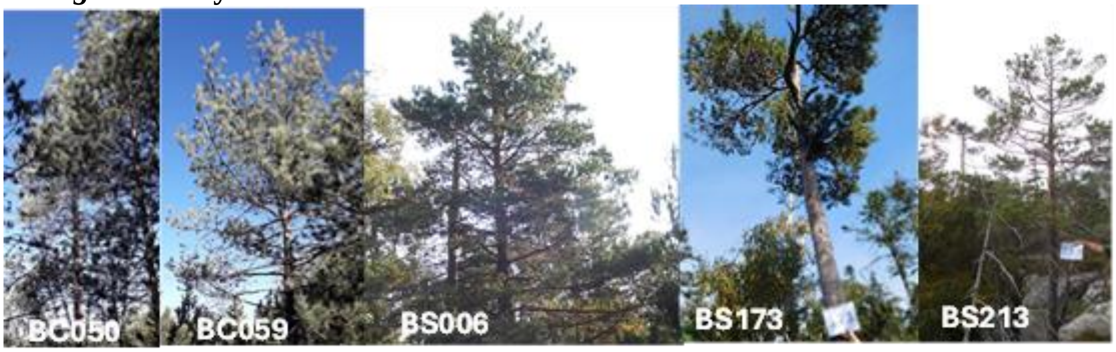

P mugo **ancestry ~0 %**

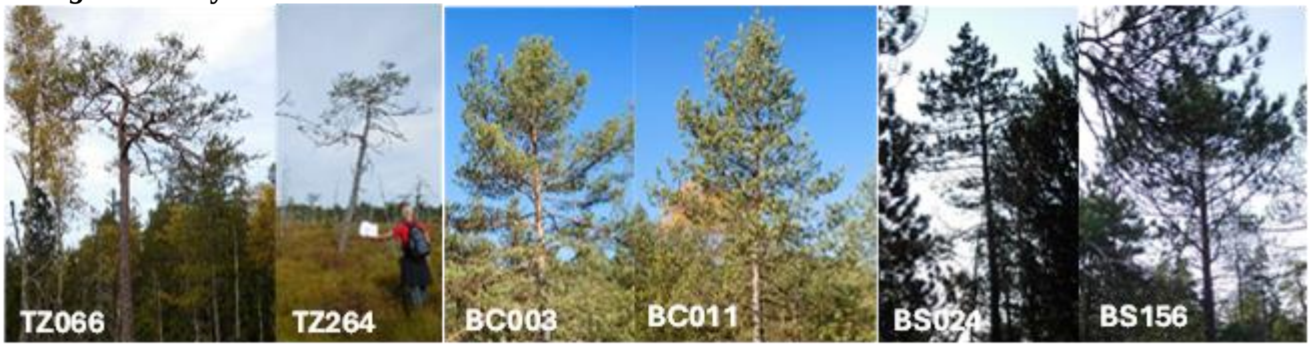

Supplement: Supplementary file 2 — Data S2: mec70137‐sup‐0002‐Data_S2.pdf. [file MEC-34-e70137-s002.pdf]
