# Supplementary material for: Repeatable Genomic Outcomes Along the Speciation Continuum: Insights From Pine Hybrid Zones (Genus Pinus)
Source: Mol Ecol. 2025 Oct 13;34(22):e70137. doi: 10.1111/mec.70137 (PMC12617033; doi:10.1111/mec.70137)
Supplement: Supplementary file 3 — Figure S1: Geographic ranges of pine species and locations of studied populations. Figure S2: Distribution of transcriptome contig mapping across retained SNPs. Figure S3: Cross‐entropy criterion from LEA analyses. Cross‐entropy values across 10 replicate runs for each K (number of clusters), ranging from 1 – 10. The optimal number of clusters is indicated by the first substantial decrease in cross‐entropy at K = 2. Figure S4: Population structure of parental species. Principal component analysis (PCA) of P. mugo and P. sylvestris individuals from allopatric populations. Individuals are colour‐coded by population as in Figure 2. Figure S5: Population structure within each hybrid zone. Principal component analysis (PCA) of individuals projected along PC1 and PC2, shown separately for each hybrid zone. Reference allopatric populations of both parental species are included for comparison. Individual trees are consistently colour‐coded by their population of origin. Allopatric populations of P. mugo and P. sylvestris are represented using gradients of their respective primary colours (as in Figure 1), while contact zone populations are shown in distinct colours. Figure S6: Genetic diversity levels in studied pines. Boxplots comparing mean values of observed heterozygosity, expected heterozygosity, allelic richness, and fixation index (F‐index) among individuals grouped by ancestry class: putative F1 (F1), later generation hybrids (H), pure Pinus mugo (PM), and pure Pinus sylvestris (PS). Figure S7: Genetic diversity metrics by population and ancestry class. Barplots showing observed heterozygosity, expected heterozygosity, allelic richness, and fixation index (F‐index) for individuals grouped by population of origin and ancestry class. Ancestry classes are colour‐coded as in Figure 3, with the addition of dark red to represent putative F1 hybrids. Figure S8: Pairwise FST differentiation between populations grouped by ancestry class. Heatmap showing pairwise FST values r [file MEC-34-e70137-s003.docx]

**Supplemental Information for:**

**Repeatable genomic outcomes along the speciation continuum: insights from pine hybrid zones (genus *Pinus*)**

Łabiszak Bartosz, Szczepański Sebastian, Wachowiak Witold

**Table of Contents:**

| **Supplemental Figures** | Page 2 |
| --- | --- |
| **Supplemental Tables** | Page 13 |

**Figure S1. Geographic ranges of pine species and locations of studied populations.**

The distribution ranges of *P. sylvestris* (light blue) and *P. mugo* (yellow) in Europe are shown. Light blue points indicate isolated populations of *P. sylvestris*. Note that the species are also separated by elevation (*P. sylvestris*: 0–1,000 m a.s.l.; *P. mugo*: 1,100–2,200 m a.s.l.), although this is not depicted here. The zoomed-in panel shows the precise locations of the studied populations, which are color-coded as in Figure 1. Species distribution data were adapted from Caudullo et al. (2024), “Chorological data for the main European woody species,” Mendeley Data, V18, https://doi.org/10.17632/hr5h2hcgg4.18.


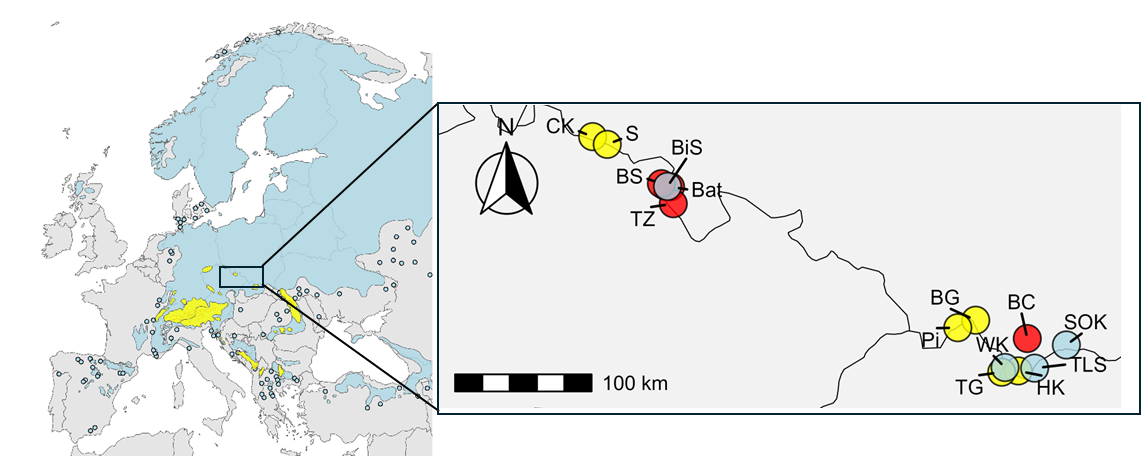


**Figure S2. Distribution of transcriptome contig mapping across retained SNPs.**

Proportion of SNP mapped to varying numbers of unique contigs, illustrating the broad genomic distribution and transcriptome-based design of the PineGAP array.

**
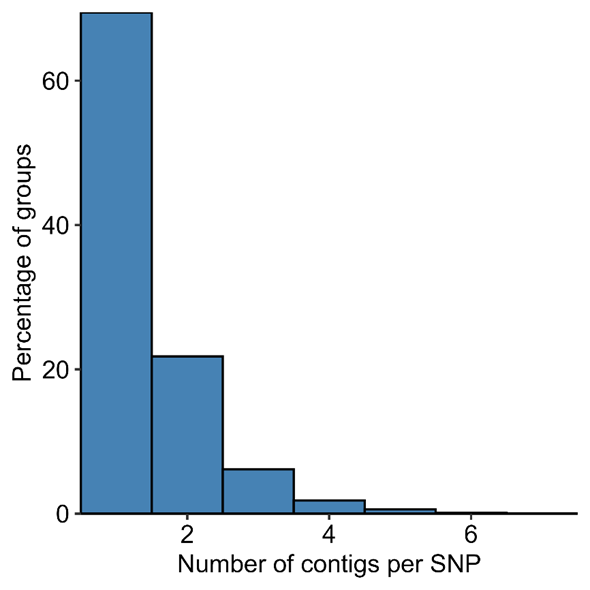
**

**Figure S3. Cross-entropy criterion from LEA analyses.** Cross-entropy values across ten replicate runs for each *K* (number of clusters), ranging from 1 to 10. The optimal number of clusters is indicated by the first substantial decrease in cross-entropy at *K* = 2.


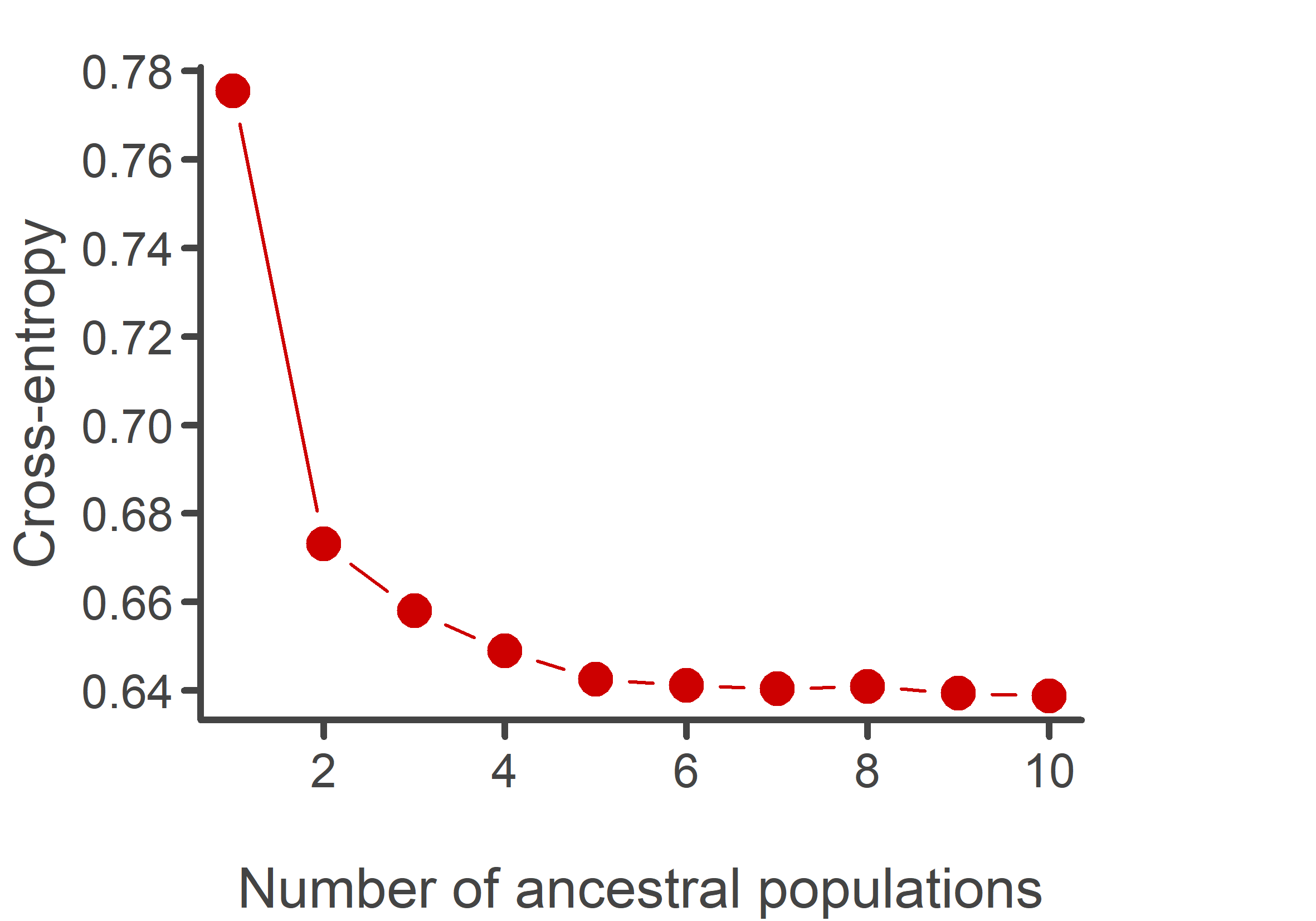


**Figure S4. Population structure of parental species.** Principal component analysis (PCA) of
*P. mugo* and *P. sylvestris* individuals from allopatric populations. Individuals are color-coded by population as in Fig. 2.


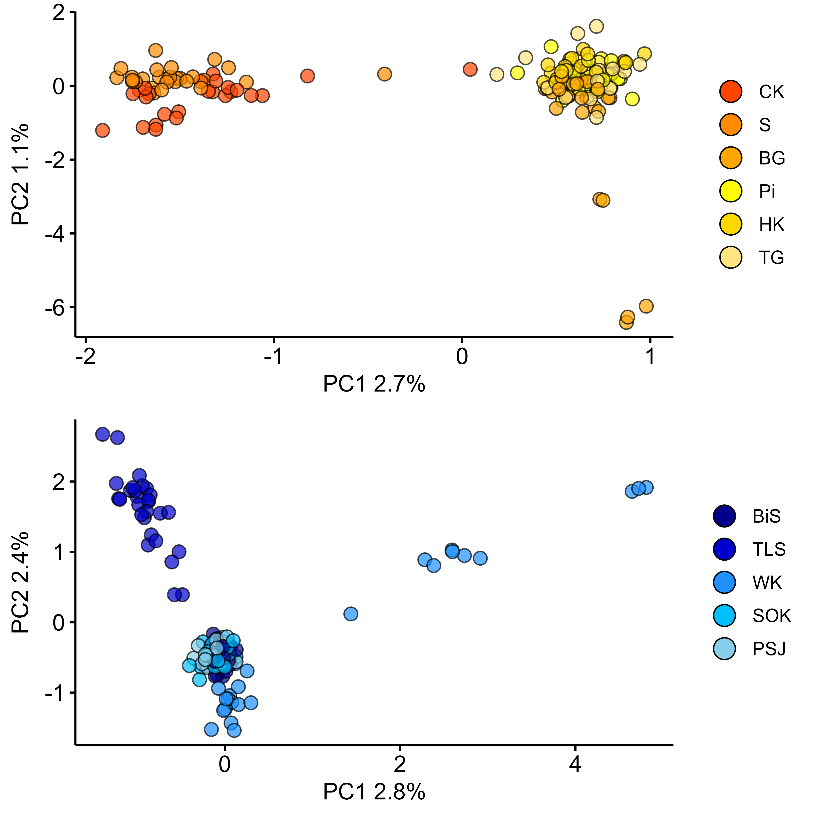


**Figure S5. Population structure within each hybrid zone.** Principal component analysis (PCA) of individuals projected along PC1 and PC2, shown separately for each hybrid zone. Reference allopatric populations of both parental species are included for comparison. Individual trees are consistently color-coded by their population of origin. Allopatric populations of *P. mugo* and *P. sylvestris* are represented using gradients of their respective primary colors (as in Fig. 1), while contact zone populations are shown in distinct colors.


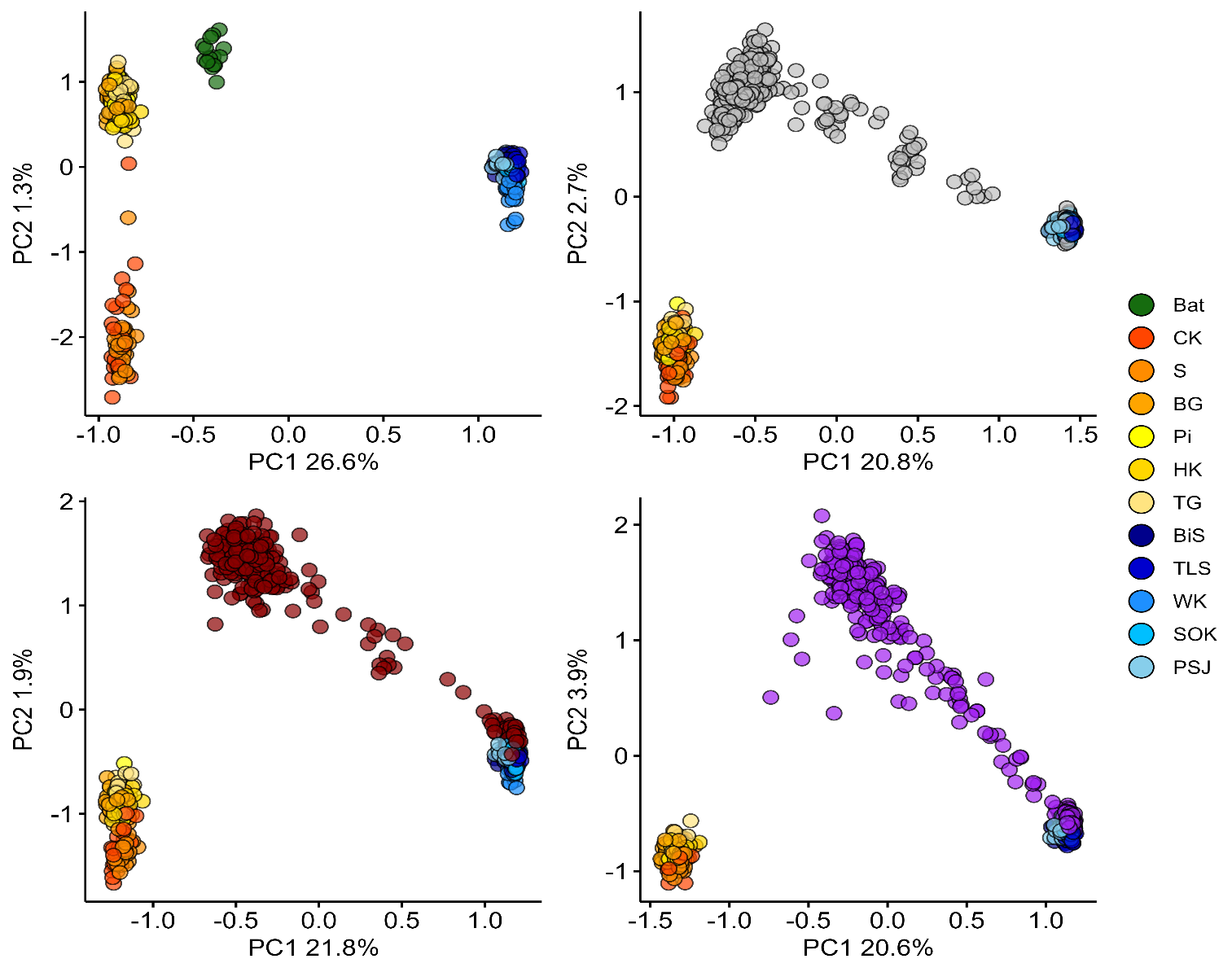

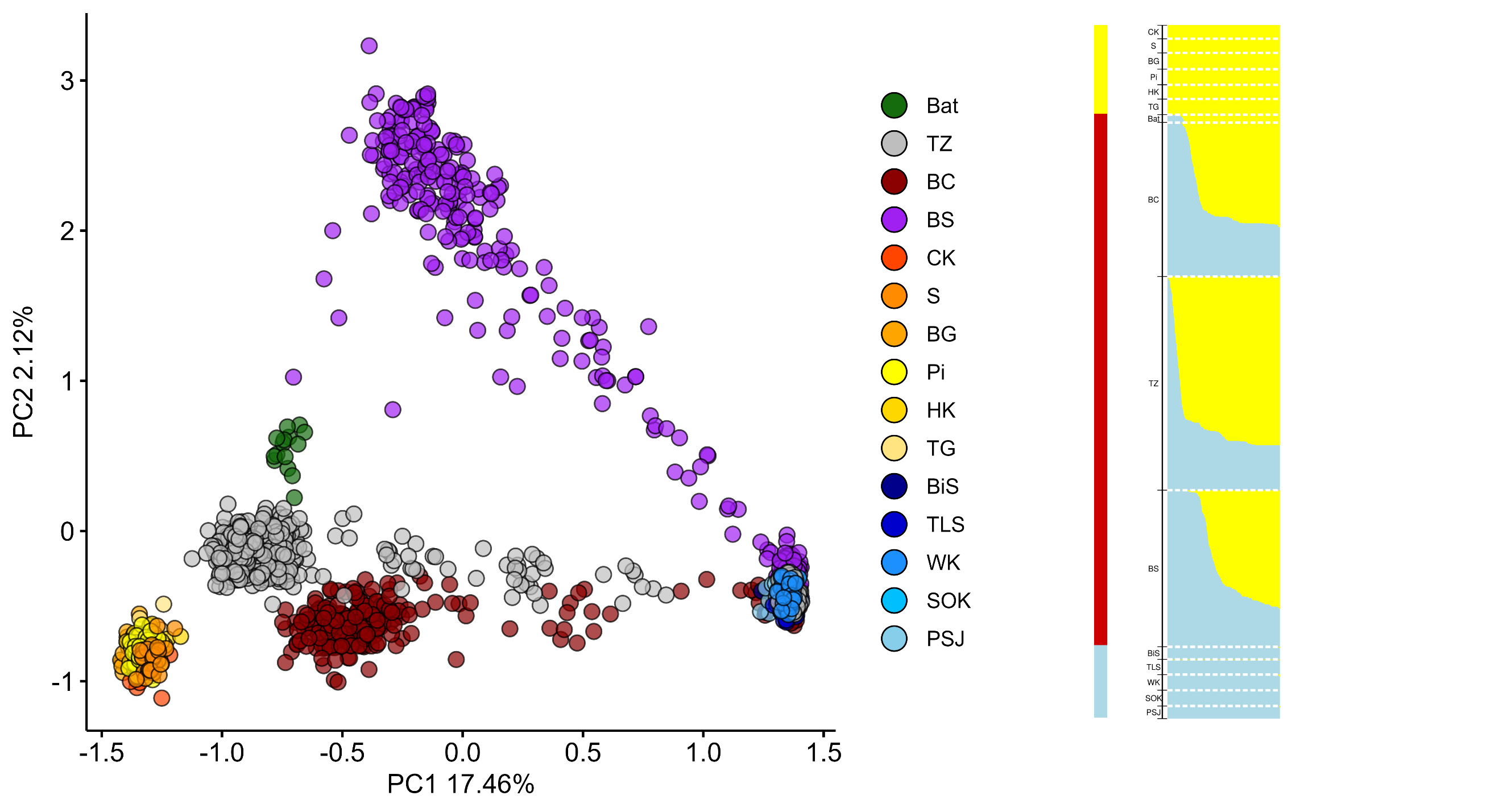


**Figure S6. Genetic diversity levels in studied pines.** Boxplots comparing mean values of observed heterozygosity, expected heterozygosity, allelic richness, and fixation index (*F*-index) among individuals grouped by ancestry class: putative F1 (F1), later generation hybrids (H), pure *Pinus mugo* (PM), and pure *Pinus sylvestris* (PS).


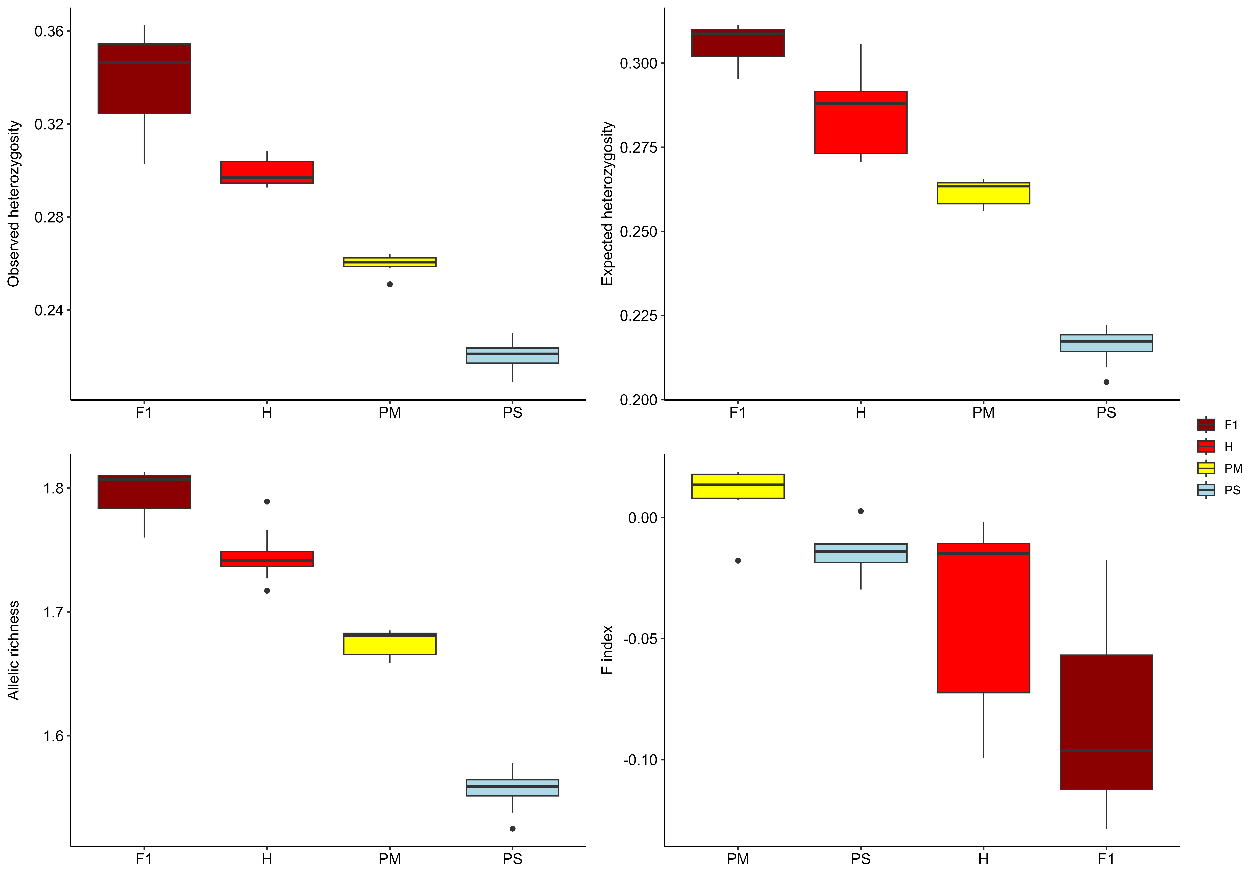


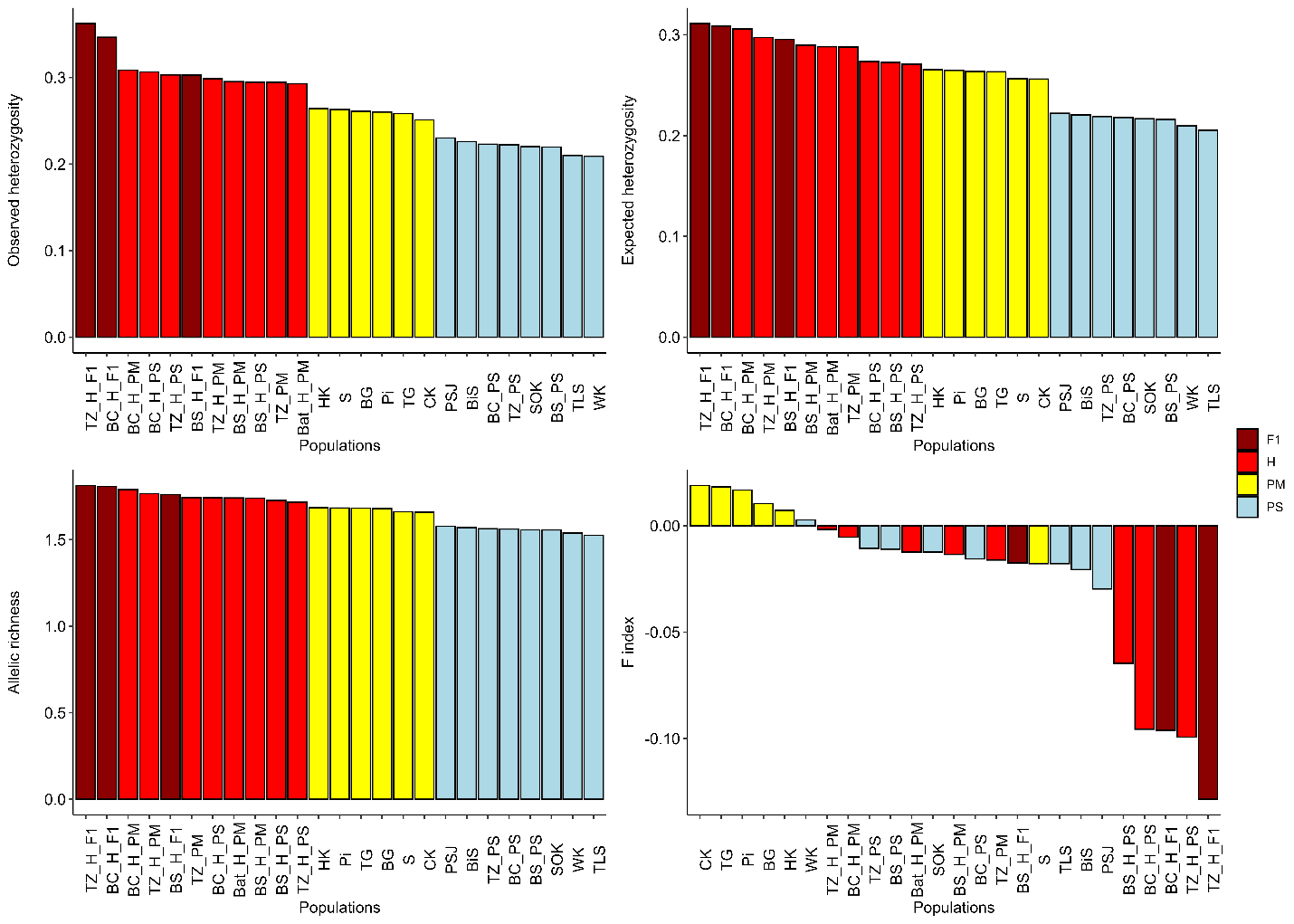
**Figure S7. Genetic diversity metrics by population and ancestry class.** Barplots showing observed heterozygosity, expected heterozygosity, allelic richness, and fixation index (F-index) for individuals grouped by population of origin and ancestry class. Ancestry classes are color-coded as in Fig. 3, with the addition of dark red to represent putative F1 hybrids.

**Figure S8. Pairwise F_ST_ differentiation between populations grouped by ancestry class**. Heatmap showing pairwise F_ST_ values representing genetic differentiation among populations. Populations are ordered and grouped according to their genetic ancestry class and geographic origin. Color intensity indicates the degree of genetic divergence, with darker shades representing higher F_ST_ values.


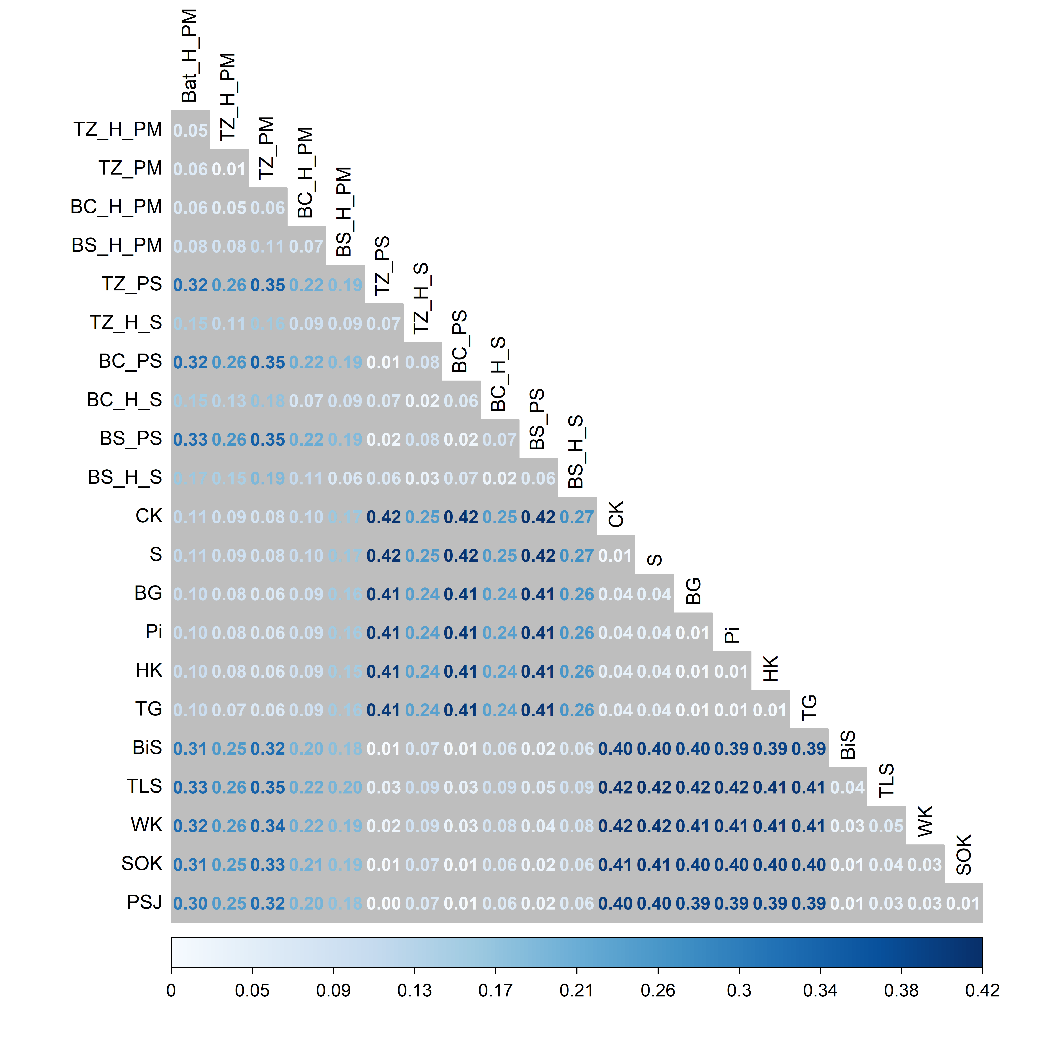

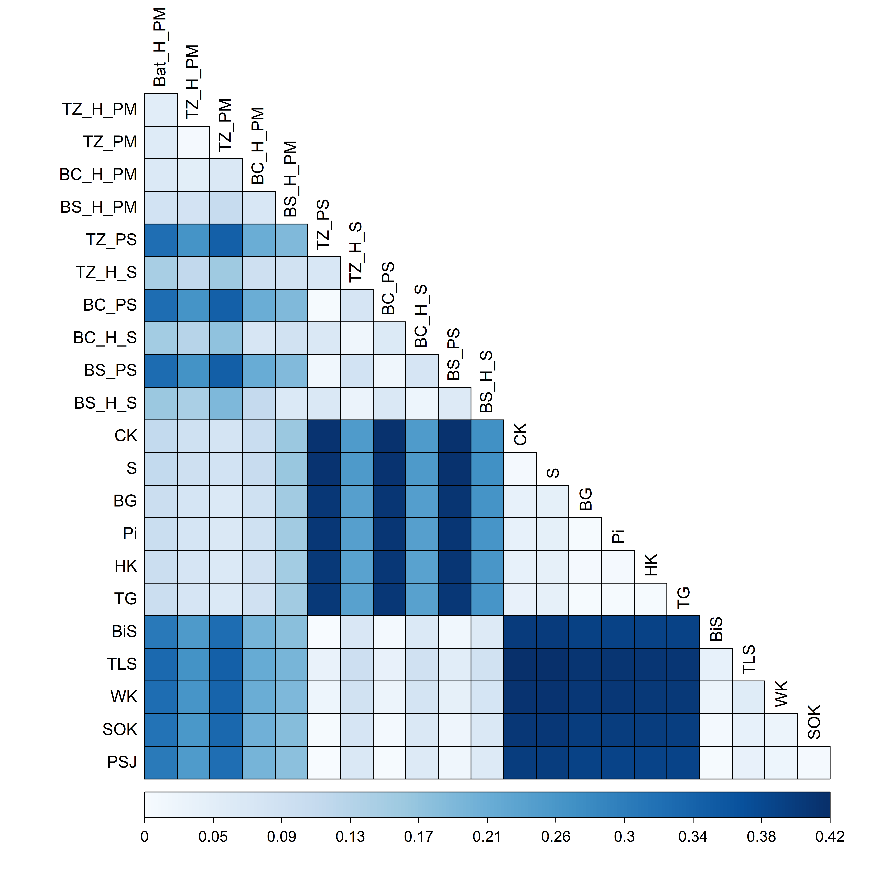


**Figure S9. Correlation between hybrid index and genomic ancestry coefficient.** Scatterplot showing the strong positive correlation between individual hybrid index values and genomic ancestry coefficients. The red regression line indicates a robust linear relationship (*R²* = 0.98, *p* < 2.2 × 10⁻¹⁶), demonstrating high concordance between the two measures of hybridization and ancestry.


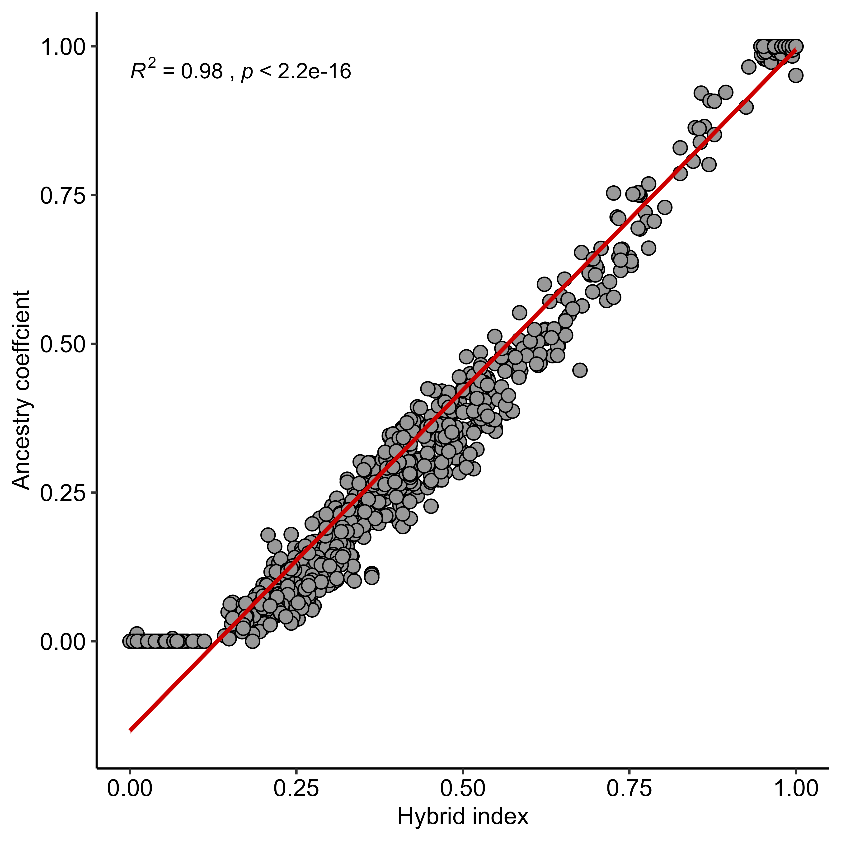


**Figure S10. Hybrid index and interclass heterozygosity across populations.**Top panel: Hybrid index values for individuals across populations, estimated using the *gghybrid* package based on ancestry-informative markers (AIMs). Individuals are color-coded by population. Dashed lines indicate thresholds for hybrid class inference. Bottom panel: Triangular plot of hybrid index versus interclass heterozygosity, generated using the *triangular* R package. This plot visualizes the distribution of individuals across hybrid classes and degrees of admixture, with color-coding matching population assignments in the top panel. Population colors correspond to those in Fig. 2.


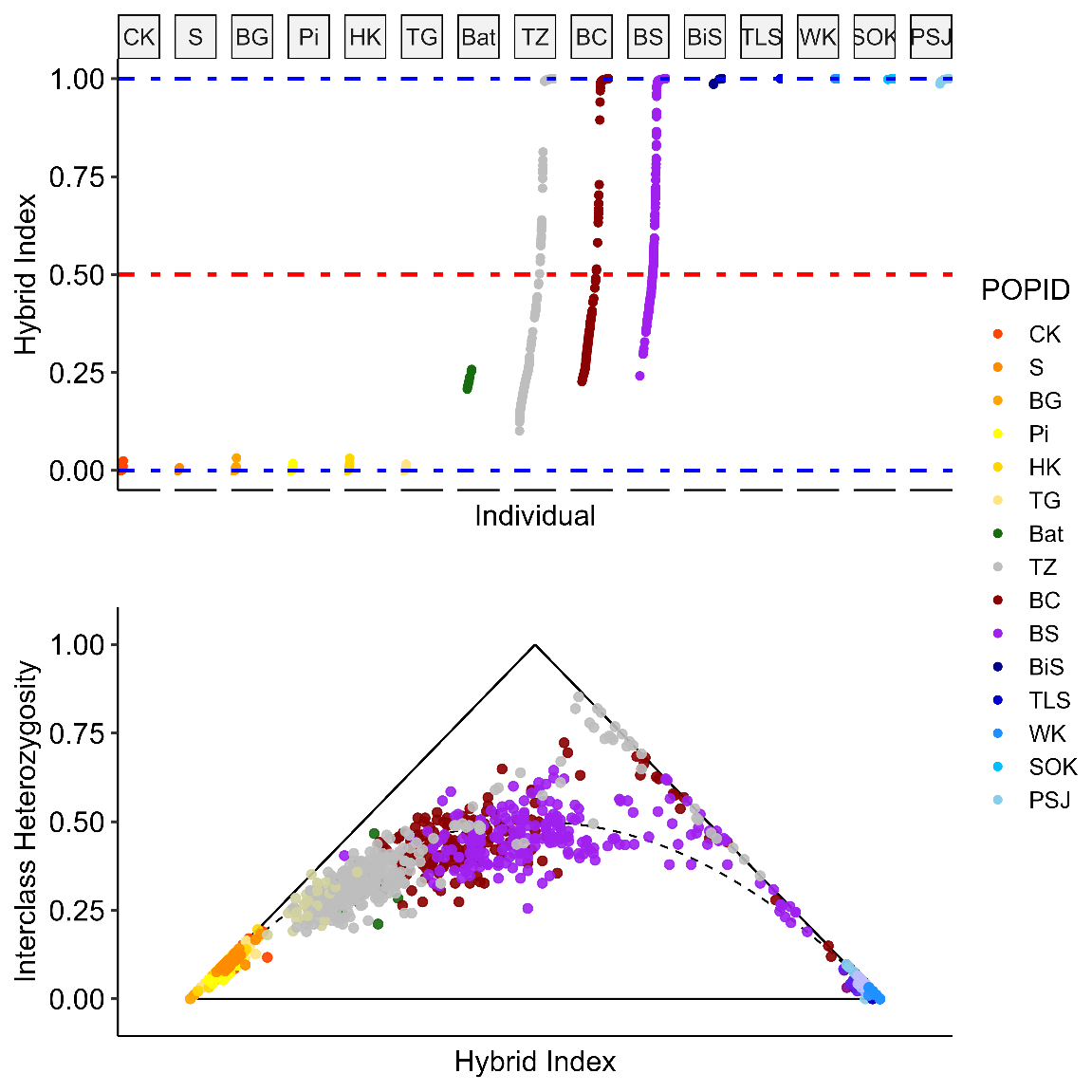


**Figure S11. Nuclear and cytoplasmic ancestry patterns across hybrid populations.**

Top panels: Principal component analysis (PCA) of individuals along PC1 (explaining 17.46% of variation), plotted by population. Individuals are color-coded by (left) nuclear ancestry class (PS, PM, H_F1, H_PM, H_PS) and (right) chloroplast DNA (cpDNA) haplotype—cp_PM (yellow) or cp_PS (light blue). Bottom panels: Proportion of cpDNA haplotypes among all hybrid individuals across the three contact zones (top: TZ, *n* = 293; BC, *n* = 195; BS, *n* = 222) and (bottom) specifically within early-generation hybrids (H_F1) (TZ, *n* = 19; BC, *n* = 11; BS, *n* = 58).


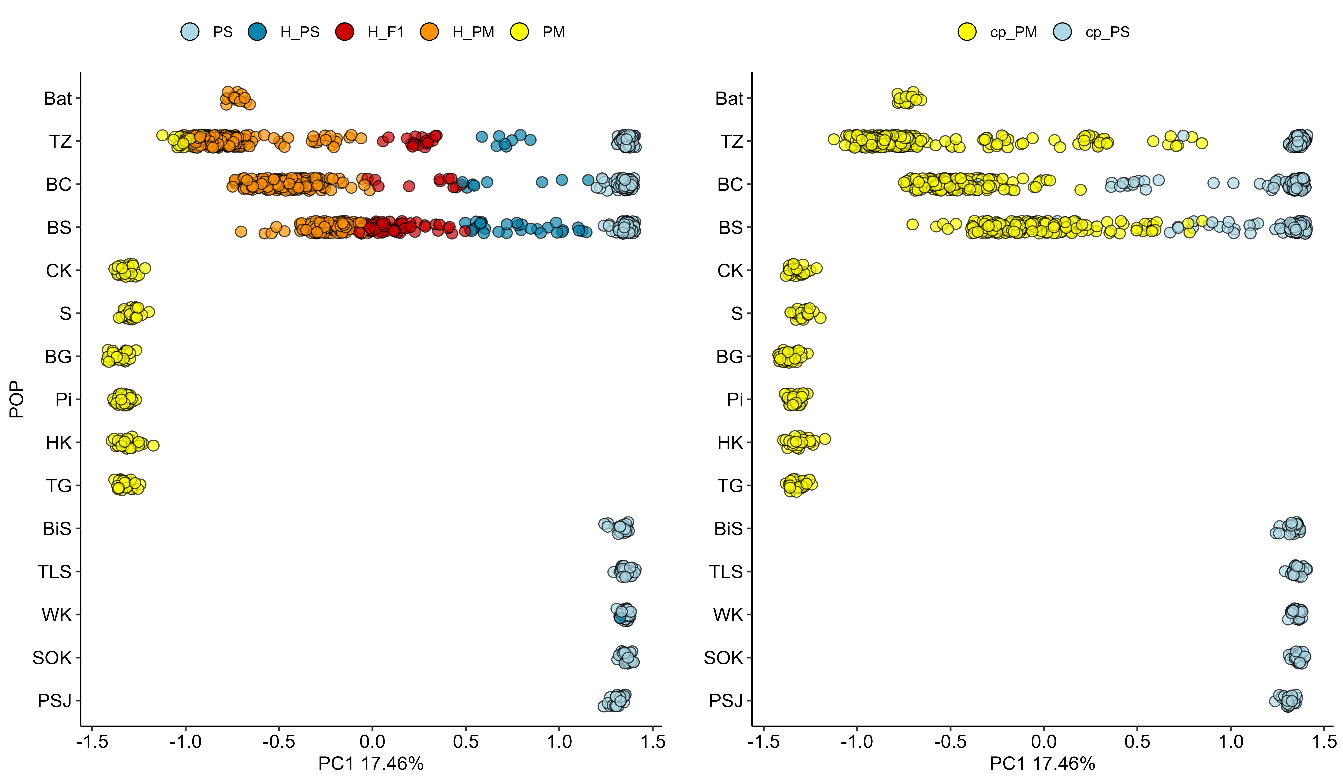

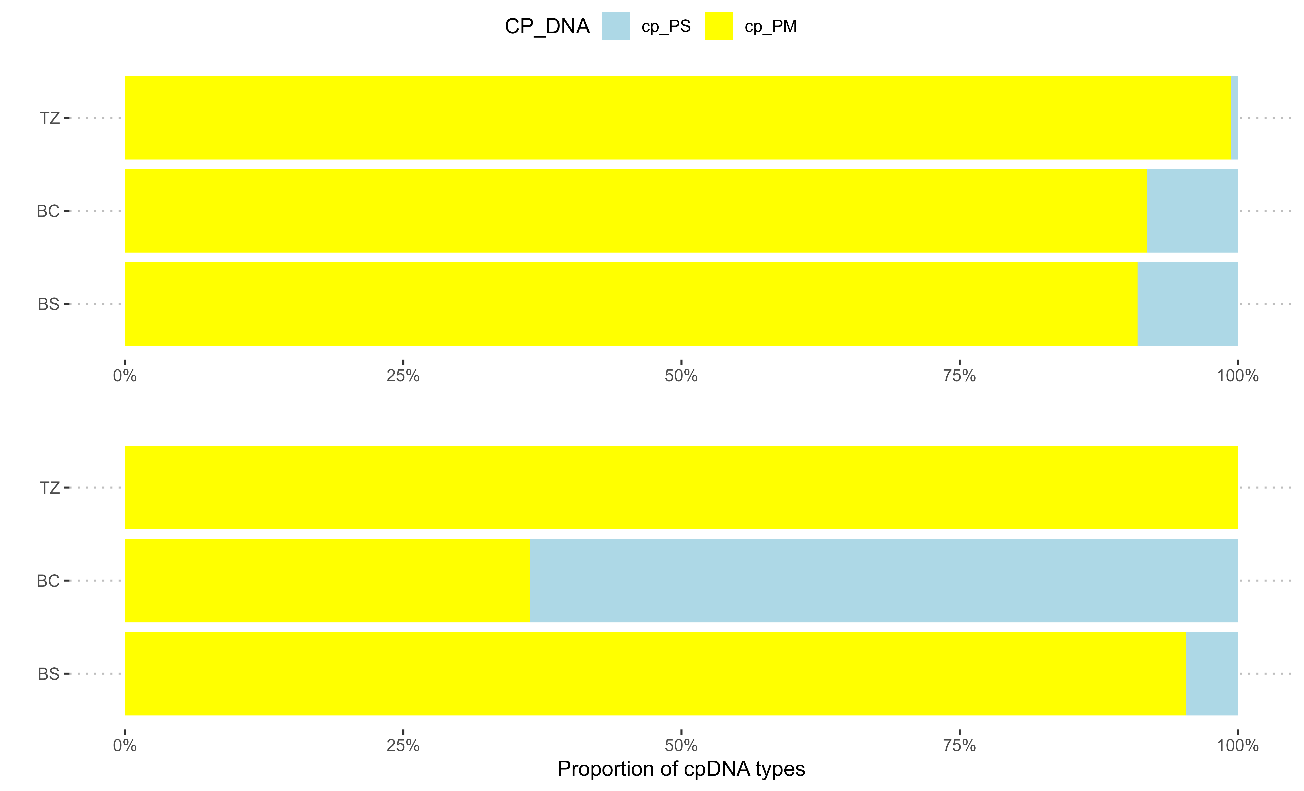


**Figure S12. Determination of the optimal number of migration edges in TreeMix analysis using an Evanno-like method.** Top: Mean log-likelihood values (± SD) across 500 TreeMix replicates for each number of migration edges (*m*, from 0 to 7), shown with the proportion of variance in the data explained (red points). The horizontal dashed line indicates the 99.8% variance threshold. Bottom: Second-order rate of change in log-likelihood (Δ*m*), calculated following an Evanno-like approach using the OptM R package. A significant drop at m = 3 indicates the most likely number of migration edges that best balances model fit and complexity.

**
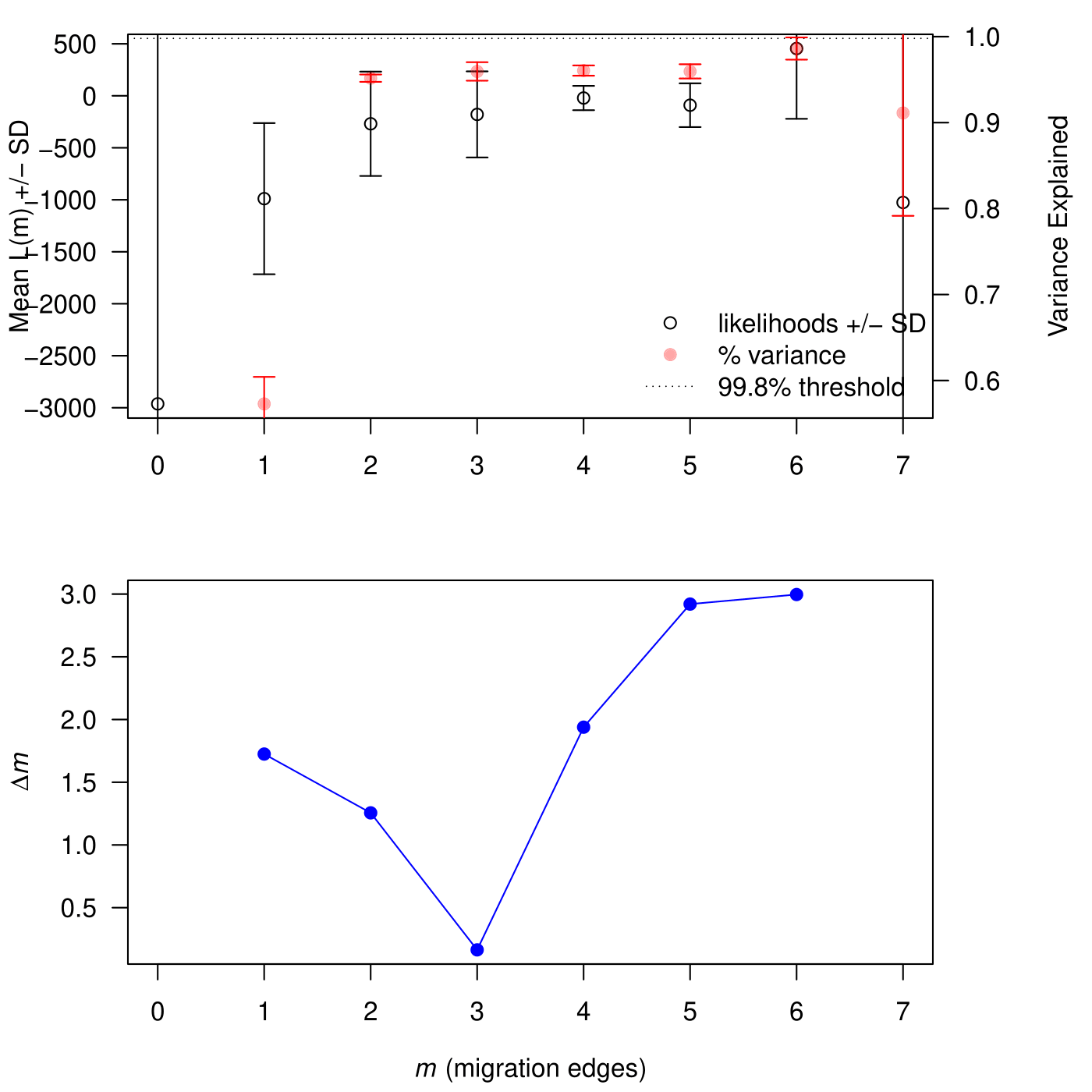
**

**Table S1. Geographic location and sample size of investigated populations.** List of hybrid and allopatric populations included in the study, with corresponding acronyms, sample sizes (*N*), population names, and geographic coordinates (longitude and latitude in WGS84). All listed individuals were used in subsequent genetic and morphological analyses.

|  | **Acronym** | **N** | **Population** | **Longitude (E)** | **Latitude (N)** |
| --- | --- | --- | --- | --- | --- |
| Hybrid populations | BAT | 15 | Wielkie Torfowisko Batorowskie | 16°23'1.68" | 50°27'29.16" |
|  | TZ | 408 | Torfowisko pod Zieleńcem | 16°24'45" | 50°20'50.28" |
|  | BC | 294 | Bór na Czerwonem | 20°2'23.64" | 49°27'37.44" |
|  | BS | 299 | Błędne Skały | 16°17'15.72" | 50°28'49.44" |
| Allopatric *P. mugo* populations | CK | 26 | Karkonosze Mountains,  Czarny Kocioł | 15°35'5.64" | 50°47'18.6" |
|  | S | 27 | Karkonosze Mountains, Śnieżka | 15°44'24.72" | 50°44'11.76" |
|  | BG | 31 | Babia Góra | 19°31'50.88" | 49°34'23.52" |
|  | Pi | 30 | Pilsko | 19°19'1.56" | 49°31'44.4" |
|  | HK | 27 | Tatra Mountains, Hala Kondratowa | 19°56'48.48" | 49°14'51" |
|  | TG | 30 | Tatra Mountains, Grześ | 19°45'59.76" | 49°14'12.12" |
| Allopatric  *P. sylvestris* populations | BiS | 24 | Stołowe Mountains, Białe Skały | 16° 21' 9.24" | 50° 27' 39.05" |
|  | TLS | 28 | Tatra Mountains, Łysa Skałka | 20°6'48.96" | 49°15'53.64" |
|  | WK | 30 | Tatra Mountains, Koryciska Wielkie | 19°48'30.24" | 49°16'10.56" |
|  | SOK | 30 | Pieniny Mountains, Sokolica | 20°26'29.04" | 49°25'6.24" |
|  | PSJ | 24 | Janów Lubelski | 22°24'42.12" | 50°42'0" |

**Table S2. ANOVA results for genetic diversity statistics among parental and hybrid groups.** Analysis of variance (ANOVA) results for allelic richness (Ar), fixation index (Fi), expected heterozygosity (He), and observed heterozygosity (Ho), comparing: (Left) three population groups — *Pinus mugo* (PM), *P. sylvestris* (PS), and hybrids (H); (Right) four groups — PM, PS, hybrids (H), and early-generation hybrids (H_F1). Significance levels: *p* ≤ 0.05 (*), *p* ≤ 0.01 (**).

| **PM – PS - H** | | | | | | **PM -PS – H – H_F1** | | | | |
| --- | --- | --- | --- | --- | --- | --- | --- | --- | --- | --- |
|  | **Df** | **Sum Sq** | **Mean Sq** | **F value** | **Pr(>F)** | **Df** | **Sum Sq** | **Mean Sq** | **F value** | **Pr(>F)** |
| Ar | 2 | 0.14574 | 0.07287 | 115.7 | 1.44e-08^**^ | 3 | 0.19539 | 0.06513 | 172.6 | 5.93e-15^**^ |
| Fi | 2 | 0.00888 | 0.00444 | 7.6 | 0.00724^**^ | 3 | 0.01859 | 0.006198 | 6.512 | 0.00275^**^ |
| He | 2 | 0.01715 | 0.00857 | 128.6 | 7.85e-09^**^ | 3 | 0.02696 | 0.008987 | 120.5 | 2.16e-13^**^ |
| Ho | 2 | 0.00985 | 0.00493 | 99.1 | 3.45e-08^**^ | 3 | 0.04098 | 0.013659 | 108.4 | 6.11e-13^**^ |

**Table S3. Tukey HSD post hoc pairwise comparisons for genetic diversity statistics.** Results of Tukey’s Honestly Significant Difference (HSD) tests for allelic richness (Ar), fixation index (Fi), expected heterozygosity (He), and observed heterozygosity (Ho), following ANOVA across:(1) three population groups — *Pinus mugo* (PM), *P. sylvestris* (PS), and hybrids (H); and
(2) four groups — PM, PS, hybrids (H), and early-generation hybrids (H_F1). Reported values include mean differences between groups, 95% confidence intervals, adjusted *p*-values, and significance levels. Significance codes: *p* ≤ 0.05 (*), *p* ≤ 0.01 (**), ns = not significant. Non-significant comparisons are additionally shaded for clarity.

| **Group** | **Statistic** | **Comparison** | **Diff** | **Lower** | **Upper** | **p adj** | **Significance** |
| --- | --- | --- | --- | --- | --- | --- | --- |
| **PM – PS - H** | Ar | PM-H | -0.11457 | -0.15778 | -0.07135 | 3.58e-05^**^ | ** |
|  | Ar | PS-H | -0.25387 | -0.29878 | -0.20896 | 0^**^ | ** |
|  | Ar | PS-PM | -0.13930 | -0.17985 | -0.09876 | 2.50e-06^**^ | ** |
|  | Ar | PS-PM | -0.11912 | -0.14837 | -0.08988 | 0^**^ | ** |
|  | Fi | PM-H | -0.03855 | -0.08006 | 0.00296 | 0.0696741 | ns |
|  | Fi | PS-H | -0.06306 | -0.10620 | -0.01992 | 0.0055335 | ** |
|  | Fi | PS-PM | -0.02451 | -0.06345 | 0.01443 | 0.2526434 | ns |
|  | Fi | PS-PM | -0.02332 | -0.06977 | 0.02312 | 0.5131785 | ns |
|  | He | PM-H | -0.04043 | -0.05450 | -0.02637 | 1.6e-05^**^ | ** |
|  | He | PS-H | -0.08725 | -0.10186 | -0.07264 | 0^**^ | ** |
|  | He | PS-PM | -0.04682 | -0.06001 | -0.03363 | 1.8e-06^**^ | ** |
|  | He | PS-PM | -0.04577 | -0.05877 | -0.03277 | 0^**^ | ** |
|  | Ho | PM-H | -0.02437 | -0.03651 | -0.01223 | 0.0004677^**^ | ** |
|  | Ho | PS-H | -0.06491 | -0.07753 | -0.05229 | 0^**^ | ** |
|  | Ho | PS-PM | -0.04054 | -0.05193 | -0.02915 | 0.0000017^**^ | ** |
|  | Ho | PS-PM | -0.03958 | -0.05648 | -0.02269 | 0.0000102^**^ | ** |
| **PM -PS – H – H_F1** | Ar | H-F1 | -0.04771 | -0.08436 | -0.01105 | 0.0079229^**^ | ** |
|  | Ar | PM-F1 | -0.11833 | -0.15662 | -0.08005 | 0.0000001^**^ | ** |
|  | Ar | PS-F1 | -0.23746 | -0.27411 | -0.20080 | 0^**^ | ** |
|  | Ar | PM-H | -0.07062 | -0.09987 | -0.04138 | 0.0000065^**^ | ** |
|  | Ar | PS-H | -0.18975 | -0.21682 | -0.16268 | 0^*^ | ** |
|  | Fi | H-F1 | 0.04213 | -0.01609 | 0.10034 | 0.2136189 | ns |
|  | Fi | PM-F1 | 0.08965 | 0.02884 | 0.15046 | 0.0026048^*^ | ** |
|  | Fi | PS-F1 | 0.06632 | 0.00811 | 0.12454 | 0.0218367^*^ | * |
|  | Fi | PM-H | 0.04752 | 0.00108 | 0.09397 | 0.0436694^*^ | * |
|  | Fi | PS-H | 0.02420 | -0.01880 | 0.06720 | 0.4168573 | ns |
|  | He | H-F1 | -0.01943 | -0.03573 | -0.00313 | 0.0157730^*^ | * |
|  | He | PM-F1 | -0.04345 | -0.06047 | -0.02643 | 0.0000029^**^ | ** |
|  | He | PS-F1 | -0.08922 | -0.10552 | -0.07292 | 0^**^ | ** |
|  | He | PM-H | -0.02402 | -0.03702 | -0.01102 | 0.0002298^**^ | ** |
|  | He | PS-H | -0.06979 | -0.08182 | -0.05775 | 0^**^ | ** |
|  | Ho | H-F1 | -0.03797 | -0.05915 | -0.01678 | 0.0003284^**^ | ** |
|  | Ho | PM-F1 | -0.07758 | -0.09971 | -0.05546 | 0^**^ | ** |
|  | Ho | PS-F1 | -0.11717 | -0.13835 | -0.09598 | 0^**^ | ** |
|  | Ho | PM-H | -0.03962 | -0.05651 | -0.02272 | 0.0000101^**^ | ** |
|  | Ho | PS-H | -0.07920 | -0.09484 | -0.06356 | 0^**^ | ** |

**Table S4. Mean pairwise F_ST_ values within and among hybrid and parental groups.** Values represent mean Weir and Cockerham’s F_ST_ estimates between genetic groups: PM – *Pinus mugo*; PS – *Pinus sylvestris* HYB – all hybrid individuals; H_PM – hybrids with majority PM ancestry; H_PS – hybrids with majority PS ancestry. These values summarize genetic differentiation within and among parental and hybrid ancestry groups.

| **Comparison** | **Mean FST** |
| --- | --- |
| Within PM | 0.0316 |
| Within PS | 0.0195 |
| Within HYB | 0.0748 |
| PM vs PS | 0.3990 |
| HYB vs PM | 0.1607 |
| HYB vs PS | 0.1703 |
| H_PM vs PM | 0.1020 |
| H_PM vs PS | 0.2450 |
| H_PS vs PM | 0.2390 |
| H_PS vs PS | 0.0710 |

**Table S5. Results of *f*₃-statistic tests for admixture among population trios.** Each row presents the outcome of a three-population test in the form *f*₃(X; A, B), where X is the target population and A and B are putative source populations. The table reports the *f*₃-statistic estimate (Est), Z-score (Z), and corresponding *p*-value (P). Significantly negative *f*₃ values (Z < –3) provide evidence of historical admixture in the target population.

| **Trio** | **Est** | **Z** | **P** | **Trio** | **Est** | **Z** | **P** | **Trio** | **Est** | **Z** | **P** |
| --- | --- | --- | --- | --- | --- | --- | --- | --- | --- | --- | --- |
| BC-BC_PS-BG | -0.0298 | -16.48 | 5.4E-61 | BC-BC_PS-CK | -0.0284 | -16.16 | 9.7E-59 | BC-BC_PS-HK | -0.0289 | -16.15 | 1.1E-58 |
| BC-BC_PS-Pi | -0.0294 | -16.34 | 5.1E-60 | BC-BC_PS-S | -0.0280 | -15.55 | 1.6E-54 | BC-BC_PS-TG | -0.0292 | -16.29 | 1.1E-59 |
| BC-BC_PS-TZ_PM | -0.0163 | -13.74 | 5.9E-43 | BC-BS_PS-BG | -0.0293 | -16.20 | 4.8E-59 | BC-BS_PS-CK | -0.0277 | -15.75 | 7.4E-56 |
| BC-BS_PS-HK | -0.0285 | -15.89 | 7.2E-57 | BC-BS_PS-Pi | -0.0289 | -16.05 | 5.5E-58 | BC-BS_PS-S | -0.0274 | -15.22 | 2.7E-52 |
| BC-BS_PS-TG | -0.0286 | -16.06 | 5.2E-58 | BC-BS_PS-TZ_PM | -0.0160 | -13.26 | 3.9E-40 | BC-BiS-BG | -0.0292 | -15.99 | 1.5E-57 |
| BC-BiS-CK | -0.0277 | -15.64 | 3.6E-55 | BC-BiS-HK | -0.0283 | -15.62 | 5.5E-55 | BC-BiS-Pi | -0.0288 | -15.80 | 3.3E-56 |
| BC-BiS-S | -0.0273 | -15.05 | 3.6E-51 | BC-BiS-TG | -0.0286 | -15.88 | 8.6E-57 | BC-BiS-TZ_PM | -0.0157 | -12.88 | 6.0E-38 |
| BC-PSJ-BG | -0.0290 | -15.70 | 1.4E-55 | BC-PSJ-CK | -0.0276 | -15.33 | 4.6E-53 | BC-PSJ-HK | -0.0283 | -15.49 | 3.8E-54 |
| BC-PSJ-Pi | -0.0287 | -15.61 | 5.9E-55 | BC-PSJ-S | -0.0272 | -14.72 | 4.8E-49 | BC-PSJ-TG | -0.0284 | -15.57 | 1.2E-54 |
| BC-PSJ-TZ_PM | -0.0158 | -13.10 | 3.2E-39 | BC-SOK-BG | -0.0296 | -16.11 | 2.2E-58 | BC-SOK-CK | -0.0280 | -15.67 | 2.4E-55 |
| BC-SOK-HK | -0.0288 | -15.82 | 2.2E-56 | BC-SOK-Pi | -0.0293 | -16.03 | 7.7E-58 | BC-SOK-S | -0.0277 | -15.11 | 1.3E-51 |
| BC-SOK-TG | -0.0290 | -16.07 | 4.1E-58 | BC-SOK-TZ_PM | -0.0160 | -13.27 | 3.6E-40 | BC-TLS-BG | -0.0298 | -16.10 | 2.5E-58 |
| BC-TLS-CK | -0.0282 | -15.83 | 1.9E-56 | BC-TLS-HK | -0.0288 | -15.59 | 7.9E-55 | BC-TLS-Pi | -0.0293 | -15.93 | 3.7E-57 |
| BC-TLS-S | -0.0279 | -15.19 | 4.2E-52 | BC-TLS-TG | -0.0289 | -15.81 | 2.9E-56 | BC-TLS-TZ_PM | -0.0162 | -13.35 | 1.1E-40 |
| BC-TZ_PS-BG | -0.0295 | -15.94 | 3.5E-57 | BC-TZ_PS-CK | -0.0280 | -15.65 | 3.3E-55 | BC-TZ_PS-HK | -0.0287 | -15.64 | 3.6E-55 |
| BC-TZ_PS-Pi | -0.0291 | -15.84 | 1.6E-56 | BC-TZ_PS-S | -0.0277 | -15.09 | 1.8E-51 | BC-TZ_PS-TG | -0.0289 | -15.81 | 2.6E-56 |
| BC-TZ_PS-TZ_PM | -0.0161 | -13.22 | 6.8E-40 | BC-WK-BG | -0.0294 | -16.08 | 3.8E-58 | BC-WK-CK | -0.0275 | -15.44 | 9.4E-54 |
| BC-WK-HK | -0.0285 | -15.73 | 8.8E-56 | BC-WK-Pi | -0.0290 | -16.00 | 1.3E-57 | BC-WK-S | -0.0272 | -14.90 | 3.3E-50 |
| BC-WK-TG | -0.0287 | -15.90 | 6.0E-57 | BC-WK-TZ_PM | -0.0157 | -12.95 | 2.3E-38 | BS-BC_PS-BG | -0.0250 | -13.32 | 1.8E-40 |
| BS-BC_PS-CK | -0.0241 | -13.14 | 2.0E-39 | BS-BC_PS-HK | -0.0243 | -13.00 | 1.3E-38 | BS-BC_PS-Pi | -0.0246 | -13.06 | 5.9E-39 |
| BS-BC_PS-S | -0.0237 | -12.68 | 7.8E-37 | BS-BC_PS-TG | -0.0242 | -12.98 | 1.5E-38 | BS-BC_PS-TZ_PM | -0.0168 | -11.68 | 1.7E-31 |
| BS-BS_PS-BG | -0.0261 | -14.39 | 6.1E-47 | BS-BS_PS-CK | -0.0250 | -14.09 | 4.3E-45 | BS-BS_PS-HK | -0.0255 | -14.08 | 5.3E-45 |
| BS-BS_PS-Pi | -0.0257 | -14.10 | 4.0E-45 | BS-BS_PS-S | -0.0247 | -13.64 | 2.2E-42 | BS-BS_PS-TG | -0.0252 | -14.06 | 6.2E-45 |
| BS-BS_PS-TZ_PM | -0.0181 | -13.05 | 6.1E-39 | BS-BiS-BG | -0.0251 | -13.51 | 1.3E-41 | BS-BiS-CK | -0.0242 | -13.28 | 3.1E-40 |
| BS-BiS-HK | -0.0244 | -13.14 | 2.0E-39 | BS-BiS-Pi | -0.0247 | -13.20 | 9.3E-40 | BS-BiS-S | -0.0238 | -12.83 | 1.1E-37 |
| BS-BiS-TG | -0.0243 | -13.21 | 8.2E-40 | BS-BiS-TZ_PM | -0.0169 | -11.80 | 3.8E-32 | BS-PSJ-BG | -0.0247 | -13.04 | 7.2E-39 |
| BS-PSJ-CK | -0.0239 | -12.81 | 1.4E-37 | BS-PSJ-HK | -0.0241 | -12.82 | 1.3E-37 | BS-PSJ-Pi | -0.0244 | -12.81 | 1.5E-37 |
| BS-PSJ-S | -0.0235 | -12.32 | 7.0E-35 | BS-PSJ-TG | -0.0240 | -12.73 | 4.3E-37 | BS-PSJ-TZ_PM | -0.0167 | -11.65 | 2.3E-31 |
| BS-SOK-BG | -0.0255 | -13.63 | 2.8E-42 | BS-SOK-CK | -0.0244 | -13.33 | 1.5E-40 | BS-SOK-HK | -0.0249 | -13.33 | 1.6E-40 |
| BS-SOK-Pi | -0.0251 | -13.40 | 5.8E-41 | BS-SOK-S | -0.0242 | -12.89 | 5.0E-38 | BS-SOK-TG | -0.0247 | -13.39 | 6.8E-41 |
| BS-SOK-TZ_PM | -0.0172 | -12.09 | 1.2E-33 | BS-TLS-BG | -0.0253 | -13.48 | 2.0E-41 | BS-TLS-CK | -0.0242 | -13.37 | 8.7E-41 |
| BS-TLS-HK | -0.0244 | -12.97 | 1.8E-38 | BS-TLS-Pi | -0.0248 | -13.18 | 1.1E-39 | BS-TLS-S | -0.0240 | -12.86 | 7.7E-38 |
| BS-TLS-TG | -0.0242 | -13.03 | 8.3E-39 | BS-TLS-TZ_PM | -0.0169 | -11.99 | 4.2E-33 | BS-TZ_PS-BG | -0.0253 | -13.35 | 1.2E-40 |
| BS-TZ_PS-CK | -0.0244 | -13.14 | 1.8E-39 | BS-TZ_PS-HK | -0.0246 | -13.04 | 7.3E-39 | BS-TZ_PS-Pi | -0.0249 | -13.10 | 3.4E-39 |
| BS-TZ_PS-S | -0.0241 | -12.73 | 4.1E-37 | BS-TZ_PS-TG | -0.0245 | -13.03 | 7.9E-39 | BS-TZ_PS-TZ_PM | -0.0172 | -11.90 | 1.2E-32 |
| BS-WK-BG | -0.0253 | -13.42 | 4.9E-41 | BS-WK-CK | -0.0240 | -12.94 | 2.6E-38 | BS-WK-HK | -0.0247 | -13.09 | 3.9E-39 |
| BS-WK-Pi | -0.0249 | -13.20 | 9.3E-40 | BS-WK-S | -0.0237 | -12.51 | 6.7E-36 | BS-WK-TG | -0.0245 | -13.08 | 4.4E-39 |
| BS-WK-TZ_PM | -0.0169 | -11.68 | 1.6E-31 | Bat-BC_PS-BG | -0.0118 | -9.27 | 1.9E-20 | Bat-BC_PS-CK | -0.0104 | -8.29 | 1.1E-16 |
| Bat-BC_PS-HK | -0.0107 | -8.46 | 2.6E-17 | Bat-BC_PS-Pi | -0.0112 | -8.66 | 4.5E-18 | Bat-BC_PS-S | -0.0104 | -7.88 | 3.3E-15 |
| Bat-BC_PS-TG | -0.0107 | -8.35 | 6.6E-17 | Bat-BC_PS-TZ_PM | 0.0006 | 0.82 | 4.1E-01 | Bat-BS_PS-BG | -0.0117 | -9.19 | 3.8E-20 |
| Bat-BS_PS-CK | -0.0102 | -8.01 | 1.1E-15 | Bat-BS_PS-HK | -0.0107 | -8.43 | 3.4E-17 | Bat-BS_PS-Pi | -0.0111 | -8.59 | 9.0E-18 |
| Bat-BS_PS-S | -0.0102 | -7.71 | 1.3E-14 | Bat-BS_PS-TG | -0.0107 | -8.32 | 9.1E-17 | Bat-BS_PS-TZ_PM | 0.0005 | 0.61 | 5.4E-01 |
| Bat-BiS-BG | -0.0119 | -9.39 | 6.2E-21 | Bat-BiS-CK | -0.0105 | -8.39 | 4.7E-17 | Bat-BiS-HK | -0.0108 | -8.64 | 5.6E-18 |
| Bat-BiS-Pi | -0.0113 | -8.79 | 1.4E-18 | Bat-BiS-S | -0.0105 | -8.01 | 1.1E-15 | Bat-BiS-TG | -0.0109 | -8.59 | 8.8E-18 |
| Bat-BiS-TZ_PM | 0.0005 | 0.58 | 5.6E-01 | Bat-PSJ-BG | -0.0117 | -9.08 | 1.1E-19 | Bat-PSJ-CK | -0.0104 | -8.14 | 4.0E-16 |
| Bat-PSJ-HK | -0.0108 | -8.44 | 3.1E-17 | Bat-PSJ-Pi | -0.0112 | -8.55 | 1.2E-17 | Bat-PSJ-S | -0.0103 | -7.67 | 1.7E-14 |
| Bat-PSJ-TG | -0.0107 | -8.26 | 1.5E-16 | Bat-PSJ-TZ_PM | 0.0005 | 0.59 | 5.5E-01 | Bat-SOK-BG | -0.0121 | -9.38 | 6.4E-21 |
| Bat-SOK-CK | -0.0105 | -8.35 | 6.6E-17 | Bat-SOK-HK | -0.0111 | -8.71 | 3.0E-18 | Bat-SOK-Pi | -0.0115 | -8.82 | 1.2E-18 |
| Bat-SOK-S | -0.0106 | -7.99 | 1.4E-15 | Bat-SOK-TG | -0.0110 | -8.61 | 7.3E-18 | Bat-SOK-TZ_PM | 0.0004 | 0.55 | 5.8E-01 |
| Bat-TLS-BG | -0.0119 | -9.11 | 7.9E-20 | Bat-TLS-CK | -0.0104 | -8.25 | 1.6E-16 | Bat-TLS-HK | -0.0107 | -8.18 | 2.9E-16 |
| Bat-TLS-Pi | -0.0112 | -8.51 | 1.8E-17 | Bat-TLS-S | -0.0104 | -7.85 | 4.0E-15 | Bat-TLS-TG | -0.0106 | -8.08 | 6.2E-16 |
| Bat-TLS-TZ_PM | 0.0007 | 0.86 | 3.9E-01 | Bat-TZ_PS-BG | -0.0117 | -9.17 | 4.9E-20 | Bat-TZ_PS-CK | -0.0104 | -8.26 | 1.4E-16 |
| Bat-TZ_PS-HK | -0.0107 | -8.42 | 3.6E-17 | Bat-TZ_PS-Pi | -0.0112 | -8.61 | 7.5E-18 | Bat-TZ_PS-S | -0.0104 | -7.88 | 3.2E-15 |
| Bat-TZ_PS-TG | -0.0107 | -8.33 | 8.1E-17 | Bat-TZ_PS-TZ_PM | 0.0006 | 0.77 | 4.4E-01 | Bat-WK-BG | -0.0120 | -9.28 | 1.8E-20 |
| Bat-WK-CK | -0.0101 | -7.92 | 2.4E-15 | Bat-WK-HK | -0.0110 | -8.52 | 1.7E-17 | Bat-WK-Pi | -0.0114 | -8.70 | 3.2E-18 |
| Bat-WK-S | -0.0102 | -7.55 | 4.2E-14 | Bat-WK-TG | -0.0109 | -8.37 | 5.6E-17 | Bat-WK-TZ_PM | 0.0006 | 0.78 | 4.4E-01 |
| TZ-BC_PS-BG | -0.0211 | -14.31 | 2.0E-46 | TZ-BC_PS-CK | -0.0194 | -13.78 | 3.5E-43 | TZ-BC_PS-HK | -0.0199 | -13.69 | 1.1E-42 |
| TZ-BC_PS-Pi | -0.0204 | -14.09 | 4.1E-45 | TZ-BC_PS-S | -0.0191 | -12.91 | 3.7E-38 | TZ-BC_PS-TG | -0.0202 | -14.12 | 2.8E-45 |
| TZ-BC_PS-TZ_PM | -0.0133 | -16.57 | 1.1E-61 | TZ-BS_PS-BG | -0.0208 | -14.20 | 8.8E-46 | TZ-BS_PS-CK | -0.0190 | -13.50 | 1.6E-41 |
| TZ-BS_PS-HK | -0.0197 | -13.63 | 2.8E-42 | TZ-BS_PS-Pi | -0.0202 | -13.96 | 2.6E-44 | TZ-BS_PS-S | -0.0187 | -12.70 | 5.6E-37 |
| TZ-BS_PS-TG | -0.0199 | -14.04 | 9.3E-45 | TZ-BS_PS-TZ_PM | -0.0133 | -16.42 | 1.3E-60 | TZ-BiS-BG | -0.0209 | -14.31 | 2.0E-46 |
| TZ-BiS-CK | -0.0193 | -13.75 | 5.2E-43 | TZ-BiS-HK | -0.0198 | -13.66 | 1.8E-42 | TZ-BiS-Pi | -0.0203 | -14.07 | 5.5E-45 |
| TZ-BiS-S | -0.0190 | -12.90 | 4.4E-38 | TZ-BiS-TG | -0.0201 | -14.20 | 9.3E-46 | TZ-BiS-TZ_PM | -0.0133 | -16.52 | 2.9E-61 |
| TZ-PSJ-BG | -0.0208 | -13.97 | 2.4E-44 | TZ-PSJ-CK | -0.0192 | -13.38 | 7.8E-41 | TZ-PSJ-HK | -0.0198 | -13.48 | 2.1E-41 |
| TZ-PSJ-Pi | -0.0203 | -13.82 | 1.8E-43 | TZ-PSJ-S | -0.0189 | -12.47 | 1.0E-35 | TZ-PSJ-TG | -0.0200 | -13.82 | 1.9E-43 |
| TZ-PSJ-TZ_PM | -0.0133 | -16.64 | 3.4E-62 | TZ-SOK-BG | -0.0212 | -14.31 | 1.9E-46 | TZ-SOK-CK | -0.0194 | -13.64 | 2.4E-42 |
| TZ-SOK-HK | -0.0201 | -13.74 | 5.8E-43 | TZ-SOK-Pi | -0.0206 | -14.15 | 1.8E-45 | TZ-SOK-S | -0.0192 | -12.82 | 1.2E-37 |
| TZ-SOK-TG | -0.0204 | -14.26 | 3.8E-46 | TZ-SOK-TZ_PM | -0.0134 | -16.74 | 6.2E-63 | TZ-TLS-BG | -0.0214 | -14.18 | 1.3E-45 |
| TZ-TLS-CK | -0.0196 | -13.67 | 1.5E-42 | TZ-TLS-HK | -0.0201 | -13.35 | 1.2E-40 | TZ-TLS-Pi | -0.0206 | -13.92 | 4.9E-44 |
| TZ-TLS-S | -0.0193 | -12.79 | 1.8E-37 | TZ-TLS-TG | -0.0202 | -13.82 | 1.9E-43 | TZ-TLS-TZ_PM | -0.0135 | -16.74 | 6.7E-63 |
| TZ-TZ_PS-BG | -0.0211 | -14.18 | 1.3E-45 | TZ-TZ_PS-CK | -0.0195 | -13.67 | 1.5E-42 | TZ-TZ_PS-HK | -0.0200 | -13.58 | 5.0E-42 |
| TZ-TZ_PS-Pi | -0.0205 | -14.01 | 1.4E-44 | TZ-TZ_PS-S | -0.0192 | -12.85 | 8.4E-38 | TZ-TZ_PS-TG | -0.0203 | -14.05 | 8.1E-45 |
| TZ-TZ_PS-TZ_PM | -0.0135 | -16.73 | 7.9E-63 | TZ-WK-BG | -0.0212 | -14.46 | 2.2E-47 | TZ-WK-CK | -0.0191 | -13.58 | 5.1E-42 |
| TZ-WK-HK | -0.0201 | -13.83 | 1.7E-43 | TZ-WK-Pi | -0.0206 | -14.30 | 2.0E-46 | TZ-WK-S | -0.0189 | -12.73 | 4.1E-37 |
| TZ-WK-TG | -0.0203 | -14.32 | 1.7E-46 | TZ-WK-TZ_PM | -0.0133 | -16.67 | 2.2E-62 |  |  |  |  |
